# Mapping influenza activity in emergency departments in France using Bayesian model‐based geostatistics

**DOI:** 10.1111/irv.12599

**Published:** 2018-08-21

**Authors:** Juliette Paireau, Camille Pelat, Céline Caserio‐Schönemann, Isabelle Pontais, Yann Le Strat, Daniel Lévy‐Bruhl, Simon Cauchemez

**Affiliations:** ^1^ Mathematical Modelling of Infectious Diseases Unit Institut Pasteur Paris France; ^2^ Centre National de la Recherche Scientifique UMR2000: Génomique évolutive, modélisation et santé (GEMS) Paris France; ^3^ Center of Bioinformatics, Biostatistics and Integrative Biology Institut Pasteur Paris France; ^4^ Santé publique France French National Public Health Agency Saint‐Maurice France

**Keywords:** geographic mapping, influenza, public health surveillance, spatial analysis

## Abstract

**Background:**

Maps of influenza activity are important tools to monitor influenza epidemics and inform policymakers. In France, the availability of a high‐quality data set from the Oscour^®^ surveillance network, covering 92% of hospital emergency department (ED) visits, offers new opportunities for disease mapping. Traditional geostatistical mapping methods such as Kriging ignore underlying population sizes, are not suited to non‐Gaussian data and do not account for uncertainty in parameter estimates.

**Objective:**

Our objective was to create reliable weekly interpolated maps of influenza activity in the ED setting, to inform Santé publique France (the French national public health agency) and local healthcare authorities.

**Methods:**

We used Oscour^®^ data of ED visits covering the 2016‐2017 influenza season. We developed a Bayesian model‐based geostatistical approach, a class of generalized linear mixed models, with a multivariate normal random field as a spatially autocorrelated random effect. Using R‐INLA, we developed an algorithm to create maps of the proportion of influenza‐coded cases among all coded visits. We compared our results with maps obtained by Kriging.

**Results:**

Over the study period, 45 565 (0.82%) visits were coded as influenza cases. Maps resulting from the model are presented for each week, displaying the posterior mean of the influenza proportion and its associated uncertainty. Our model performed better than Kriging.

**Conclusions:**

Our model allows producing smoothed maps where the random noise has been properly removed to reveal the spatial risk surface. The algorithm was incorporated into the national surveillance system to produce maps in real time and could be applied to other diseases.

## 1 INTRODUCTION

At least three million people are severely affected by seasonal influenza each year, leading to substantial morbidity and mortality and inducing important stress on healthcare structures.^1^ Seasonal influenza can result in patient overload in secondary healthcare settings, in particular hospital emergency departments (EDs). During these epidemics, it is essential for healthcare authorities to have at their disposal an accurate representation of the localized risk of influenza, ideally in real time, to wisely adjust the healthcare offer by increasing bed capacity, reallocating human resources or postponing nonurgent care. As part of a surveillance toolbox, maps of influenza activity could prove useful in such context. Disease maps provide a visual summary of complex geographic information, to facilitate interpretation of the data, to highlight existing patterns across space and to identify areas of elevated risk.^2^ Beyond their importance for surveillance and decision‐making, disease maps can also be interesting communication tools towards clinicians, partners, healthcare managers and the general public. Because of their visual and intuitive appeal, maps released in official surveillance reports are often published by mainstream media during outbreaks.^3^, ^4^

Generating reliable disease maps requires two important features: the availability of high‐quality data and the use of sound statistical methods. Epidemiological surveillance is often based on sentinel systems, in which a network of selected general practitioners (GPs) or healthcare facilities report cases. Sentinel surveillance systems have made it possible to considerably improve our understanding of epidemic dynamics. For instance, surveillance data from the French Sentinelles network was used to quantify the impact of school closure on influenza epidemics.^5^ However, some challenging aspects remain while working with sentinel data, such as the small proportion of participating GPs, irregular reporting and coarse spatial resolution. The availability of new types of standardized data on hospital ED visits offers new opportunities. In France, the Oscour^®^ network, a syndromic surveillance system, represents a complementary source of disease surveillance data, covering 92% of all emergency hospital visits in the country, with automatic and near real‐time transmission of individual‐level data.^6^ It does not monitor influenza in the general population but makes it possible to obtain a very detailed picture of influenza activity in secondary health care, by providing high‐quality data with larger volume, higher spatial resolution and fewer reporting delays.

When working with spatial point data, the objective of disease mapping is often to predict the continuous (ie, interpolated) risk surface of the disease over the study region, where the noise has been properly filtered. Due to the complex nature of spatial data and management of uncertainty in the estimates, the generation of such maps requires the use of appropriate statistical methods. In geostatistics, the most widely used tool, known as Kriging, allows to carry out spatial interpolation or smoothing of observed values, by constructing a linear predictor for unobserved values of a continuous spatial process and estimating the covariance structure of the data with a tool known as the variogram.^7^, ^8^ However, traditional Kriging is less appropriate when considering non‐Gaussian outcomes (eg, disease counts or proportions) and does not account fully for inherent uncertainties, such as those arising from the uneven distribution of underlying population and those associated with estimating the variogram parameters.^9^ Despite these limitations, Kriging is still used to map influenza activity.^10^, ^11^, ^12^

We propose to rely on an alternative statistical approach, Bayesian model‐based geostatistics (MBG), a class of generalized linear mixed models, with a multivariate normal random field as a spatially autocorrelated random effect. Our objective was to create reliable weekly interpolated maps of influenza activity in the ED setting in France using emergency hospital data from the Oscour^®^ network, to inform Santé publique France, the French national public health agency, and local healthcare authorities. We developed an algorithm to routinely produce weekly maps that can be used as surveillance, decision‐making and communication tools, and integrated it into MASS, a web application used at Santé publique France for monitoring influenza activity.^13^ Although this model was developed for influenza in France, the methodological framework we describe in this study could be more widely applied, both to other diseases and to other surveillance systems.

## 2 DATA

Following the 2003 heat wave in France, Santé publique France set up a new syndromic surveillance system, which included the Oscour^®^ network (“Organisation de la Surveillance Coordonnée des Urgences,” Coordinated Health Surveillance of Emergency Departments), based on hospital EDs. The creation of this network was motivated by the need to provide high‐quality information to public health authorities to help with evidence‐based decision‐making and to have a real‐time assessment of the situation in secondary health care.^14^ This surveillance network has already been described elsewhere.^6^, ^13^ Briefly, data are collected directly from patients’ computerized medical files filled in during medical consultations. For each patient, the collected data include date, age, gender, zip code, ED identification number, reason for emergency visit, main and associated medical diagnosis based on the tenth edition of the International Classification of Diseases (ICD‐10), and whether the patient was admitted for hospitalization after discharge. Encrypted data are transmitted daily to Santé publique France. All hospital discharge records are anonymous and are processed in line with national patient confidentiality rules.

The number of hospital EDs participating in the network regularly increased over time, from 23 in its creation in 2004 to 688 in 2017. It covered 92% of all hospital visits in France in 2017, with at least one ED per administrative district (French “départements”) on the metropolitan territory (5.8 on average per district) and a mean daily volume of about 50 000 visits. As a case study, we used data from 7 November 2016 to 2 April 2017, covering the 2016‐2017 season of influenza epidemic in France. The study focused on metropolitan France. Details on geographic data are provided in Section 1 in Appendix S1.

We included influenza‐coded cases, which gathered visits with ICD‐10 codes J09, J10 and J11 as clinical diagnosis. We used, as a measure of risk, the weekly proportion of influenza‐coded cases among all coded visits, which comprises visits with all diagnostic types: diseases, accidents, injuries, etc. This indicator allowed us to account for the variability in the volumes of visits among EDs and over time. We used the number of coded visits as a denominator rather than the total number of visits (coded and noncoded), so that the measured risk is not biased by the proportion of coding, which can vary over space and time (Section 2 in Appendix S1).

## 3 MODEL

### 3.1 Modelling framework

Our objective was to create weekly continuous surfaces of influenza activity in metropolitan France, using point‐referenced data at each ED locations. Geostatistics capitalize on the spatial correlation between observations to carry out spatial interpolation or smoothing of the attribute of interest, filtering the noise in the observations and highlighting existing patterns. The basis of our approach is a body of theory known as Bayesian MBG.^15^ MBG combine the efficiency of classical geostatistical interpolation algorithms for spatial prediction with the formalization and flexibility of generalized linear modelling and allow the application of Bayesian methods of statistical inference for parameter estimation and spatial prediction.^16^ Uncertainty is rigorously handled at all stages of the modelling process.

### 3.2 Model description

In the geostatistical framework, the point‐referenced data are realizations of an underlying spatial process (or random field) {*U*(*s*)*, s *∈ *D*} characterized by a spatial index *s* which varies continuously in the fixed domain *D*. Here, the location *s* was a two‐dimensional vector with latitude and longitude. For each week, we assumed that our observations (number of influenza‐coded cases) available at *n* spatial locations and represented by the vector *y *= (*y*(*s*_1_),…, *y*(*s*_*n*_)), where the set (*s*_1_,…, *s*_*n*_) indicates the locations of EDs, followed a binomial distribution:

$$y\left(s_{i}\right)∼B\left(N_{i},p_{i}\right)$$

with *N*_*i*_ the total number of coded visits in location *i* and *p*_*i*_ the influenza probability for location *i*. The linear predictor was defined as the logistic transformation of *p*_*i*_ and included an intercept α, a random effect represented by *U*(*s*_*i*_) which is the realization of a random field *U* at the location *s*_*i*_ and an unstructured random error (residual noise) *e*_*i*_:

$$\text{logit}\left(p_{i}\right)=\alpha +U\left(s_{i}\right)+e_{i}$$

The component $e_{i}$ was modelled as Gaussian with zero mean and variance $\sigma_{e}^{2}$. The random field was modelled as a Gaussian field (GF), so that the vector (*U*(*s*_*1*_),…,*U*(*s*_*n*_)) followed a multivariate normal distribution with zero mean and spatially structured covariance matrix Σ:

U(s)∼MVN(0,Σ)

The covariance matrix Σ was defined by the Matérn spatial covariance function:

$$\Sigma_{\mathrm{ij}}=\text{Cov}\left(U\left(s_{i}\right),U\left(s_{j}\right)\right)=\;\frac{\sigma^{2}}{\Gamma \left(\lambda \right)2^{\lambda -1}}{\left(\kappa \|s_{i}-s_{j}\|\right)}^{\lambda }K_{\lambda }\left(\kappa \right\|s_{i}-s_{j}\left\|\right)$$

where ‖ *s*_*i*_ ‐ *s*_*j*_ ‖  is the Euclidean distance between two locations *s*_*i*_ and *s*_*j*_ and *σ* ^2^ is the marginal variance. The term *K*_*λ*_ denotes the modified Bessel function of the second kind and order *λ > *0. The parameter *λ* measures the degree of smoothness of the process and is usually kept fixed due to poor identifiability. Conversely, *κ > *0 is a scaling parameter related to the spatial range *r*, that is the distance at which the spatial correlation becomes almost null (ie, <0.1) via the empirically derived definition $r\approx \frac{\sqrt{8\lambda }}{\kappa }$.^17^ Bayesian specification was then completed by assigning prior distributions to parameters and hyperparameters (Section 3 in Appendix S1).

### 3.3 Implementation

The model was implemented using the integrated nested Laplace approximation (INLA) method introduced by Rue et al,^18^ which provides fast and reliable calculations of posterior marginal distributions, avoiding time‐consuming MCMC simulations. First, the model was fitted to the ED data and the parameters of the spatial model were estimated. Then, the posterior mean and standard deviation for the response variable were predicted at each pixel of a regular grid covering France. We used a grid of 2 km resolution (572 × 530 pixels) to obtain a smooth posterior surface. Every week was fitted independently. All stages were coded using the R‐INLA package (http://www.r-inla.org). Model theory and implementation in R have already been thoroughly described.^18^, ^19^, ^20^ We provide a summary of key features and the R code for the model in Section 3 in Appendix S1. Two complementary maps were generated: in the first map, we used the posterior mean of the proportion of influenza‐coded cases as a point estimate for each pixel, while in the second map, we represented the coefficient of variation (relative standard deviation) to highlight areas with more or less uncertainty. The coefficient of variation is a standardized measure of dispersion of a probability distribution and is defined as the ratio of the standard deviation to the mean. It shows the extent of variability in relation to the mean.

### 3.4 Model assessment

First, to assess how well the MBG model fitted the data, we represented the observed values against the fitted values at the observation level (ED), and computed Pearson's linear correlation coefficient. Second, to further assess the accuracy of the spatial prediction method, we compared the observed proportion of influenza‐coded cases at the district level (N = 96) to the grid predictions averaged by district. Three statistics were computed: Pearson's linear correlation coefficient, the mean error (same unit as the data), which indicates whether the predictions are biased by being on average too low or too high, and the mean absolute error, which measures the average magnitude of prediction errors. Third, we checked that our model was not overfitting the data, by comparing the predictions made by the full model (ie, the model with all observations) at the ED locations, to the predictions made when each observation is, in turn, removed from the model (leave‐one‐out predictions). If the predictions are close, it means that the interpolated surface is not too sensitive to the data and that the model is able to predict the smoothed proportion of influenza‐coded cases in an unsampled location. We compared the MBG results with those obtained using Kriging, the traditional method for spatial interpolation and smoothing of point data. Details can be found in Section 4 in Appendix S1.

## 4 RESULTS

**Figure 1:**
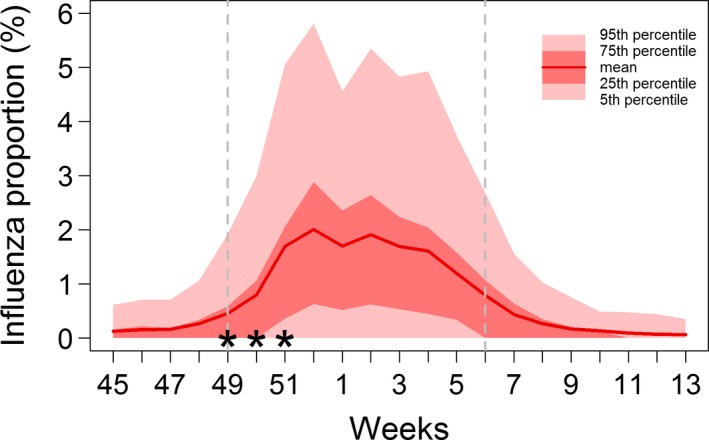
Weekly proportion of influenza‐coded cases among all coded visits by hospital emergency departments of the Oscour^®^ network during the 2016‐2017 influenza season in France. Stars show the 3 weeks for which detailed results and maps are presented. The dashed grey lines delimit the epidemic period.

Over the study period (7 November 2016‐2 April 2017) in metropolitan France, 5 589 477 visits were coded in the Oscour^®^ database, representing 74.1% of all visits (coded and noncoded). Among the coded visits, 0.82% (N = 45 565) were classified as influenza cases. The influenza epidemic lasted 10 weeks, from week 49 of 2016 (December 5‐11) to week 6 of 2017 (February 6‐12) (Figure 1).^21^

**Figure 2:**
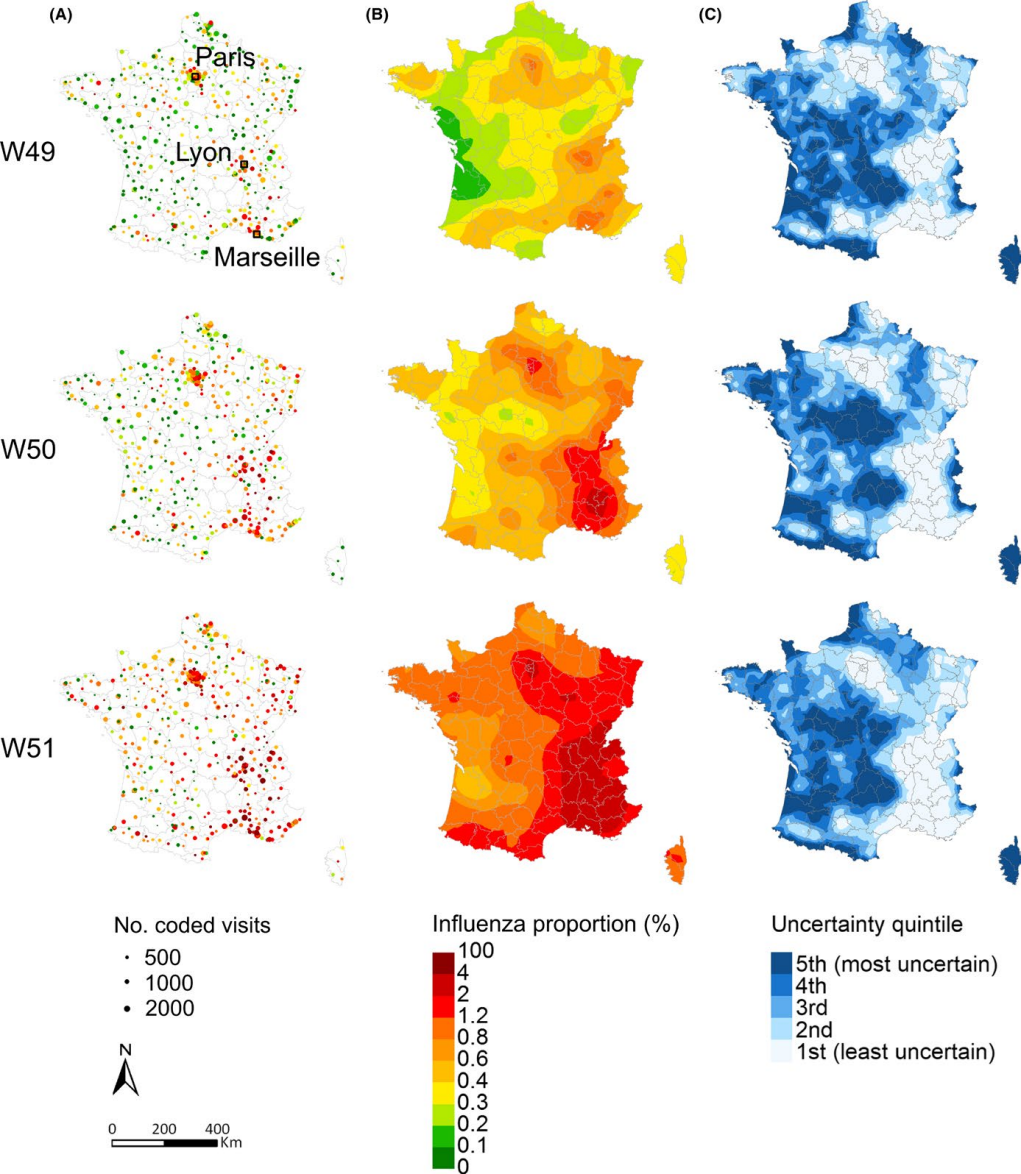
Maps for weeks 49, 50 and 51 of 2016. A, Observed proportion of influenza‐coded cases at each ED locations; B, Posterior mean of predicted proportion on the 2 × 2 km grid; C, Relative uncertainty associated with the predicted proportion, quantified using the coefficient of variation and ordered into quintiles such that areas in quintile one have the smallest uncertainty and quintile five the largest. Grey borders delimit administrative districts (N = 96).

We present detailed results for 3 weeks during the ascending phase of the epidemic (December 5‐25, 2016), when having good estimations in real time is the most important for decision‐makers (Figure 1). Among the 645 EDs which provided data through the Oscour^®^ surveillance network during these 3 weeks, 509 (78.9%) coded at least one influenza case. The mean number of coded visits per ED per week was 462 (interquartile range [IQR] 212‐649). The mean proportion of influenza‐coded cases among all coded visits increased from 0.45% in week 49 to 0.80% in week 50 and 1.69% in week 51 (Figure 1). Figure 2 presents the maps resulting from our geostatistical model for these 3 weeks. The maps for the entire study period are provided in Section 5 in Appendix S1. Over the season, the highest proportions were mostly observed in densely populated regions, around Lyon and Marseille in the southeast quarter of France, followed by the Parisian region (Figure 2 and Section 5 in Appendix S1). The epidemic started in these more urbanized regions and then quickly expanded to the whole country. The map of the coefficient of variation allows highlighting the areas where the relative uncertainty in the predictions was the highest. These were mostly areas in the centre of France, as well as along borders, with fewer data points and/or low number of visits in sparsely populated areas. The uncertainty was lower in urban areas with higher concentrations of populations and hospitals. Posterior means and 95% credible intervals for the model parameters are provided in Table 1. The posterior mean of the range was 168 km for week 49, indicating that the spatial correlation became negligible beyond this distance. It increased to 176 and 227 km for weeks 50 and 51, respectively.

**Table 1:** Posterior mean (95% credible interval) of the model's parameters for 3 weeks in December 2016

Parameter	Week 49	Week 50	Week 51
Intercept, *α*	−6.50 (−6.32, −5.81)	−5.55 (−5.84, −5.30)	−4.70 (−4.94, −4.47)
Standard deviation of the noise, *σ*_*e*_	0.74 (0.62, 0.88)	0.71 (0.61, 0.81)	0.51 (0.39, 0.68)
Spatial range of the GF, *r* (km)	168 (81, 326)	176 (94, 310)	227 (119, 411)
Marginal standard deviation of the GF, *σ*	0.57 (0.40, 0.77)	0.64 (0.47, 0.91)	0.62 (0.56, 0.70)

**Figure 3:**
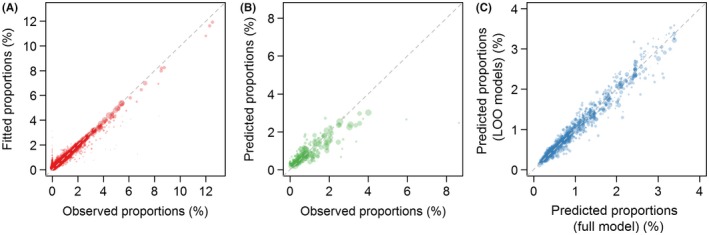
Model assessment for weeks 49‐51. A, Scatterplot of fitted and observed proportions of influenza‐coded cases, at each ED locations. For scale reasons, one outlier was not represented on the graph: observed proportion of 100% (1 influenza‐coded case among 1 coded visit) for a predicted proportion of 5%; B, Scatterplot of predicted and observed proportions, averaged at the district level; C, Scatterplot of predictions of the full model at ED locations and the leave‐one‐out predictions. Point size is weighted by the number of all coded visits. The dashed line is the bisector.

Figure 3 presents the three scatterplots used for model assessment. Regarding the fit of the MBG model (inference stage), the Pearson correlation coefficient between the observed and fitted values at the ED locations for the 3 weeks was 0.94, indicating excellent linear agreement (Figure 3A). Regarding the spatial predictions, when comparing the observed and predicted mean proportions at the district level, the Pearson correlation coefficient was 0.80 and the mean prediction error was −0.00016, in units of the influenza proportion (−0.016%), indicating no systematic bias in the predicted rates (Figure 3B). The mean absolute error was 0.0032. The mean proportion of influenza‐coded cases predicted by the model at the district level was 0.84%, with an IQR between 0.39 and 1.06, compared to 0.85% with an IQR between 0.29 and 1.15 for the observed proportion. This indicates a shrinkage of extreme values towards the global mean, an expected result due to spatial smoothing. Finally, the Pearson correlation coefficient between the predictions of the full model at the ED locations and the leave‐one‐out predictions was 0.98, with a mean error of 0.0007 and a mean absolute error of 0.0012 (Figure 3C). Overall, our model performed better than the standard Kriging method (Section 4 in Appendix S1). In particular, Kriging predictions at the district level underestimated influenza activity (mean error of ‐0.25%, 15 times higher than the MBG mean error) and the correlation coefficient for the leave‐one‐out predictions at the ED locations was lower (*r *=* *0.84) than with the MBG model.

## 5 DISCUSSION

Disease maps are more and more often used in infectious diseases epidemiology, as they are efficient surveillance and control tools. However, due to the complex nature of spatial data and management of uncertainty in the estimates, the generation of such maps must be performed with care to provide an unbiased representation of disease spatial patterns. Here, we have developed a Bayesian geostatistical model to create weekly maps of influenza activity in the ED setting in metropolitan France. Our model allows producing smoothed maps where the random noise has been properly removed to reveal the spatial risk surface, and is not constrained by administrative boundaries. This makes the interpretation of the data easier. The model has been incorporated into the national syndromic surveillance system, as described below. We provided the R code so that the model can be more widely applied to other countries or diseases.

Spatial interpolation of disease rates can be performed using a variety of simple techniques, including inverse distance‐weighted methods, splines or trend polynomial surfaces. The main limitations of these deterministic methods is that they do not provide a measure of the reliability of the predictions and do not take advantage of the spatial structure of the variable.^10^, ^22^ In 1992, Carrat and Valleron were the first to apply Kriging to the spatial analysis of an infectious disease. At that time, Kriging was a real improvement over the existing approaches as it made use of the spatial correlation between observations and allowed for estimation of the interpolation error.^10^ However, traditional Kriging is not well suited to the analysis of disease rates as it does not take into account the underlying population size, while rates computed from sparsely populated areas tend to be less reliable,^22^ and works best for data that follow a normal distribution, which is hardly fulfilled with counts or proportions.^23^ Indeed, non‐Gaussian observations can affect the variogram estimate and lead to incorrect conclusions, as shown by our supporting analysis. Although trans‐Gaussian Kriging can be used to overcome this issue,^7^ we showed that, in our case, the Kriging predictions were less accurate than the MBG predictions and that Kriging may underestimate uncertainty in the predictions. Uncertainty attached to model parameters is indeed ignored in the analysis, which typically leads to too small prediction variances.^24^ To overcome the issues associated with standard Kriging, we chose to use a Bayesian model‐based geostatistical approach. Other alternatives have been proposed, such as using Poisson kriging^22^ or assessing variogram uncertainty in a Bayesian framework.^25^ Bayesian geostatistical models such as the one developed in this study are appropriate for non‐Gaussian data, by specifying an explicit stochastic model, and yield the full posterior distribution of the risk while accounting for the uncertainty in the model parameters (such as the shape of the covariance function). They have been shown to be valuable methodologies for generating predictive prevalence and risk maps for malaria,^16^, ^26^ shistosomiasis^27^ or poverty,^28^ among others. In our model, the spatial dependence was defined using a Matérn covariance function. We observed that the spatial range *r*, a parameter of this covariance function, was increasing during the ascending phase of the epidemic, as the epidemic spread through the country and the affected areas become larger. As spatial correlation is mainly due to population connectivity between locations, parameters of a mechanistic model explicitly describing flows of individuals would be fixed over time. But here, this underlying mechanism is approximated with a phenomenological model, leading *r* to vary over time.

Beyond the implementation of an appropriate statistical methodology, generating reliable disease maps also requires the availability of high‐quality data. In this study, we have used the Oscour^®^ network, which gathers daily data from ED visits in France. The data are automatically extracted from the computerized medical files that are filled by clinicians as part of their routine activities, and transmitted every day, avoiding the reporting delays that can impair other surveillance systems, without requiring additional efforts from clinicians provided that they have a business software for emergency medicine. This gives a very detailed picture of the daily situation in secondary health care all over the country, this information being of great importance for healthcare managers. Although this surveillance might have suffered from limited spatial representativeness in some regions during its first years of existence,^29^ the network now includes 92% of all visits and gives a satisfactory coverage of the metropolitan territory in all regions. As our model is able to produce good estimates in unsampled locations, we are confident that our maps are representative of the spatial risk surface in all regions, with good spatial resolution.

In this study, we used as a risk indicator the proportion of influenza‐coded cases among all coded visits, as previously carried out by others.^30^, ^31^ This allowed us to account for the variability in the volumes of visits among EDs, and the proportion of coding, which can vary over space and time (Section 2 in Appendix S1). One limitation of this measure of risk is its sensitivity to the “case mix,” that is the variability in the number of noninfluenza cases, which can bias the proportion of influenza‐coded cases if unusual variations are observed. The number of noninfluenza cases did not display large temporal variations over the study period and therefore should not substantially affect the observed trends (Section 2 in Appendix S1). However, we cannot rule out that the proportion of influenza‐coded cases might be impacted by the spatial variations in the case mix. The ICD‐10 codes used to classify cases as influenza are not perfectly sensitive nor specific to influenza but we have no reason to think that coding practices significantly differed among EDs. In addition, the proportion of influenza‐coded cases is a mixture of the true influenza risk in the general population and the care‐seeking behaviours, which depend on (a) the severity of the virus (ED surveillance systems tend to capture more severe cases), (b) the ease of access to an ED (the risk might be underestimated in areas where access to EDs is more difficult, due to lower density of health services and longer travel time to hospitals) and (c) the socioeconomic status (the risk might be overestimated in neighbourhoods with lower socioeconomic status that have no access to a GP). Thus, our maps must be primarily seen as the spatial representation of the influenza risk in the ED setting. This data set is complementary to another influenza surveillance system, the Sentinelles network of volunteer GPs, which monitors influenza consultations in general practice and produces maps using Kriging.^10^ It has been shown that data from both sources followed similar temporal patterns.^6^ Spatial patterns, however, are not expected to be directly comparable. First, they do not measure the same risk and populations are different regarding age distribution and severity profiles.^13^ Second, the Oscour^®^ network generates a larger volume of data (number of cases is five times higher than in the Sentinelles network) and has a better spatial resolution (on average ~650 participating EDs by week, compared to ~260 GPs participating in the Sentinelles network by week).^32^ Third, the Sentinelles maps are produced by applying Kriging to incidence estimated at the district level (ie, only 96 spatial points), without taking into account their precision (which depends on the number of participating GPs by district).^10^

For our results to efficiently support surveillance and be accessible to public health professionals, it is essential that they are integrated in an effective information flow where data are gathered, analysed and reported on a weekly basis. This is performed through the intranet online application, called MASS, which has been developed by Santé publique France to provide their epidemiologists with an easy access to up‐to‐date surveillance data from specific and syndromic surveillance systems and to the results of statistical analyses of the epidemiologic risk.^10^ One aim of this study was to complete this surveillance tool by developing automated disease mapping. The algorithm that we developed in R was thus added to MASS so that weekly disease maps can now be visualized. Along with the smoothed influenza activity map, the algorithm output also provides an uncertainty map. Indeed, it is important that scientists better communicate about uncertainties in model estimates as this might potentially affect interpretation of the data. Uncertainty in our results arises from at least four sources: low density of data sources in some areas, low volume of visits in some EDs, uncertainty in the spatial parameters and inherent spatial heterogeneity in influenza proportions that occurs over short spatial scale and that cannot be explained by the data and modelling approaches. This latter component of variation is captured as “noise” (randomness) by the geostatistical model and the smoothing process causes the loss of local details of the spatial variation in risk. But the model ensures that the degree of randomness is measured and incorporated in the predicted posterior distributions at each pixel.^16^

This framework can be the basis for future developments, to include the temporal dimension, as well as population movements and connectivity for instance. Our algorithm could also be easily applied to other countries or any other diseases monitored by a surveillance system where cases are geographically referenced. Although our approach was mainly descriptive, with the aim of supporting surveillance, further research should aim at developing methods for predicting influenza activity in the ED setting in order to support local healthcare planning.

## References

[1] WHO . Influenza (seasonal) fact sheet. http://www.who.int/mediacentre/factsheets/fs211/en/. Accessed June 27, 2017.

[2] Elliott P , Wartenberg D . Spatial epidemiology: current approaches and future challenges. Environ Health Perspect. 2004;112:998‐1006. 15198920 10.1289/ehp.6735 PMC1247193

[3] L'Internaute . Grippe 2017 : symptômes, vaccin, traitement… Tout savoir sur l’épidémie. http://www.linternaute.com/actualite/societe/1353255-grippe-le-pic-de-l-epidemie-n-est-pas-encore-atteint-apprenez-a-reperer-les-symptomes/. Accessed June 27, 2017.

[4] NBC News . Flu season is off to a strong start, CDC says. http://www.nbcnews.com/health/health-news/flu-season-getting-worse-cdc-says-n704061. Accessed August 21, 2017.

[5] Cauchemez S , Valleron A‐J , Boëlle P‐Y , Flahault A , Ferguson NM . Estimating the impact of school closure on influenza transmission from Sentinel data. Nature. 2008;452:750‐754. 18401408 10.1038/nature06732

[6] Josseran L , Nicolau J , Caillère N , Astagneau P , Brücker G . Syndromic surveillance based on emergency department activity and crude mortality: two examples. Euro Surveill. 2006;11:225‐229. 17370967

[7] Cressie NAC . Statistics for Spatial Data. New York, NY: John Wiley & Sons, Inc; 2013.

[8] Matheron G . Principles of geostatistics. Econ Geol. 1963;58:1246‐1266.

[9] Pullan RL , Sturrock HJW , Soares Magalhães RJ , Clements ACA , Brooker SJ . Spatial parasite ecology and epidemiology: a review of methods and applications. Parasitology. 2012;139:1870‐1887. 23036435 10.1017/S0031182012000698 PMC3526959

[10] Carrat F , Valleron AJ . Epidemiologic mapping using the ‘kriging’ method: application to an influenza‐like illness epidemic in France. Am J Epidemiol. 1992;135:1293‐1300. 1626546 10.1093/oxfordjournals.aje.a116236

[11] Sakai T , Suzuki H , Sasaki A , Saito R , Tanabe N , Taniguchi K . Geographic and temporal trends in influenza‐like illness, Japan, 1992–1999. Emerg Infect Dis. 2004;10:1822‐1826. 15504270 10.3201/eid1010.040147 PMC3323282

[12] Uphoff H , Stalleicken I , Bartelds A , Phiesel B , Kistemann BT . Are influenza surveillance data useful for mapping presentations? Virus Res. 2004;103:35‐46. 15163486 10.1016/j.virusres.2004.02.010

[13] Pelat C , Bonmarin I , Ruello M , et al. Improving regional influenza surveillance through a combination of automated outbreak detection methods: the 2015/16 season in France. Euro Surveill. 2017;22:30593. 28816649 10.2807/1560-7917.ES.2017.22.32.30593 PMC6373610

[14] Caserio‐Schönemann C , Bousquet V , Fouillet A , Henry V , pour l’équipe projet SurSaUD® . The French syndromic surveillance system SurSaUD^®^ . Bull Epidemiol Hebd. 2014;3–4:38‐44.

[15] Diggle PJ , Ribeiro PJ . Bayesian inference in Gaussian model‐based geostatistics. Geogr Environ Model. 2002;6:129‐146.

[16] Gething PW , Patil AP , Smith DL , et al. A new world malaria map: *Plasmodium falciparum* endemicity in 2010. Malar J. 2011;10:378. 22185615 10.1186/1475-2875-10-378 PMC3274487

[17] Lindgren F , Rue H , Lindström J . An explicit link between Gaussian fields and Gaussian Markov random fields: the stochastic partial differential equation approach: link between Gaussian fields and Gaussian Markov random fields. J R Stat Soc Ser B Stat Methodol. 2011;73:423‐498.

[18] Rue H , Martino S , Chopin N . Approximate Bayesian inference for latent Gaussian models by using integrated nested Laplace approximations. J R Stat Soc Ser B Stat Methodol. 2009;71:319‐392.

[19] Blangiardo M , Cameletti M . Spatial and Spatio‐Temporal Bayesian Models with R‐INLA. Chichester: John Wiley and Sons, Inc.; 2015 308 p.

[20] Martins TG , Simpson D , Lindgren F , Rue H . Bayesian computing with INLA: new features. Comput Stat Data Anal. 2013;67:68‐83.

[21] Équipes de surveillance de la grippe . Surveillance de la grippe en France, saison 2016‐2017. Bull Epidémiol Hebd. 2017;22:466‐475.

[22] Ali M , Goovaerts P , Nazia N , Haq MZ , Yunus M , Emch M . Application of Poisson kriging to the mapping of cholera and dysentery incidence in an endemic area of Bangladesh. Int J Health Geogr. 2006;5:45. 17038192 10.1186/1476-072X-5-45 PMC1617092

[23] Magalhães RJS , Clements ACA , Patil AP , Gething PW , Brooker S . The applications of model‐based geostatistics in helminth epidemiology and control. Adv Parasitol. 2011;74:267‐296. 21295680 10.1016/B978-0-12-385897-9.00005-7 PMC3037997

[24] Goovaerts P , Gebreab S . How does Poisson kriging compare to the popular BYM model for mapping disease risks? Int J Health Geogr. 2008;7:6. 18248676 10.1186/1476-072X-7-6 PMC2276482

[25] Marchant BP , Lark RM . The Matérn variogram model: implications for uncertainty propagation and sampling in geostatistical surveys. Geoderma. 2007;140:337‐345.

[26] Alegana VA , Atkinson PM , Lourenço C , et al. Advances in mapping malaria for elimination: fine resolution modelling of *Plasmodium falciparum* incidence. Sci Rep. 2016;6:srep29628. 10.1038/srep29628 PMC4942778 27405532

[27] Vounatsou P , Raso G , Tanner M , N'goran EK , Utzinger J . Bayesian geostatistical modelling for mapping schistosomiasis transmission. Parasitology. 2009;136:1695‐1705. 19490724 10.1017/S003118200900599X

[28] Steele JE , Sundsøy PR , Pezzulo C , et al. Mapping poverty using mobile phone and satellite data. J R Soc Interface. 2017;14:pii: 20160690. 10.1098/rsif.2016.0690 PMC5332562 28148765

[29] Josseran L , Caillère N , Goncalves N , et al. Surveillance syndromique dans le cadre de la pandémie grippale A(H1N1)2009 : intérêts et limites. Bull Epidémiologique Hebd. 2010;24–26:274‐277.

[30] Olson DR , Heffernan RT , Paladini M , Konty K , Weiss D , Mostashari F . Monitoring the impact of influenza by age: emergency department fever and respiratory complaint surveillance in New York City. PLoS Med. 2007;4:e247. 17683196 10.1371/journal.pmed.0040247 PMC1939858

[31] Olson DR , Konty KJ , Paladini M , Viboud C , Simonsen L . Reassessing Google flu trends data for detection of seasonal and pandemic influenza: a comparative epidemiological study at three geographic scales. PLoS Comput Biol. 2013;9:e1003256. 24146603 10.1371/journal.pcbi.1003256 PMC3798275

[32] Institut Pierre Louis d'Epidémiologie et de Santé Publique (UMR S 1136) . Bilan annuel 2016, Réseau Sentinelles. 2016.

