# Supplementary material for: Mapping influenza activity in emergency departments in France using Bayesian model‐based geostatistics
Source: Influenza Other Respir Viruses. 2018 Aug 21;12(6):772–9. doi: 10.1111/irv.12599 (PMC6185885; doi:10.1111/irv.12599)
Supplement: Supplementary file 1 [file IRV-12-772-s001.docx]

**Appendix S1**

**Mapping influenza activity in emergency departments in France using Bayesian model-based geostatistics**

Juliette Paireau, Camille Pelat, Céline Caserio-Schönemann, Isabelle Pontais, Yann Le Strat, Daniel Lévy-Bruhl, Simon Cauchemez

Contents

[1. Geographic data 1](#_Toc521855290)

[2. Percentage of coding 2](#_Toc521855291)

[3. Methods for the Bayesian geostatistical model 2](#_Toc521855292)

[3.1. Model theory 2](#_Toc521855293)

[3.2. Priors 4](#_Toc521855294)

[3.3. R code 5](#_Toc521855295)

[4. Comparison to Kriging 7](#_Toc521855296)

[5. Weekly maps for the 2016-2017 season 10](#_Toc521855297)

[6. References 16](#_Toc521855298)

# 1. Geographic data

The geographic coordinates (latitude and longitude) of all emergency departments (EDs) were downloaded from the open platform for French public data (URL: www.data.gouv.fr/fr/datasets/extraction-du-fichier-national-des-etablissements-sanitaires-et-sociaux-finess-par-etablissements/) and merged with the Oscour® dataset.

The shapefile of administrative districts was obtained from the GEOFLA® database of the National Institute of Geographic and Forestry Information (URL: professionnels.ign.fr/geofla).

The study focused on metropolitan France, using the Lambert93 projection system, based on the RGF93 geodetic system. When two ED had the same coordinates (e.g. two services in the same hospital), they were merged together so that every pair of coordinates was unique in the dataset.

# 2. Percentage of coding

Figure S1 presents data on the coding of ED visits over time and space.


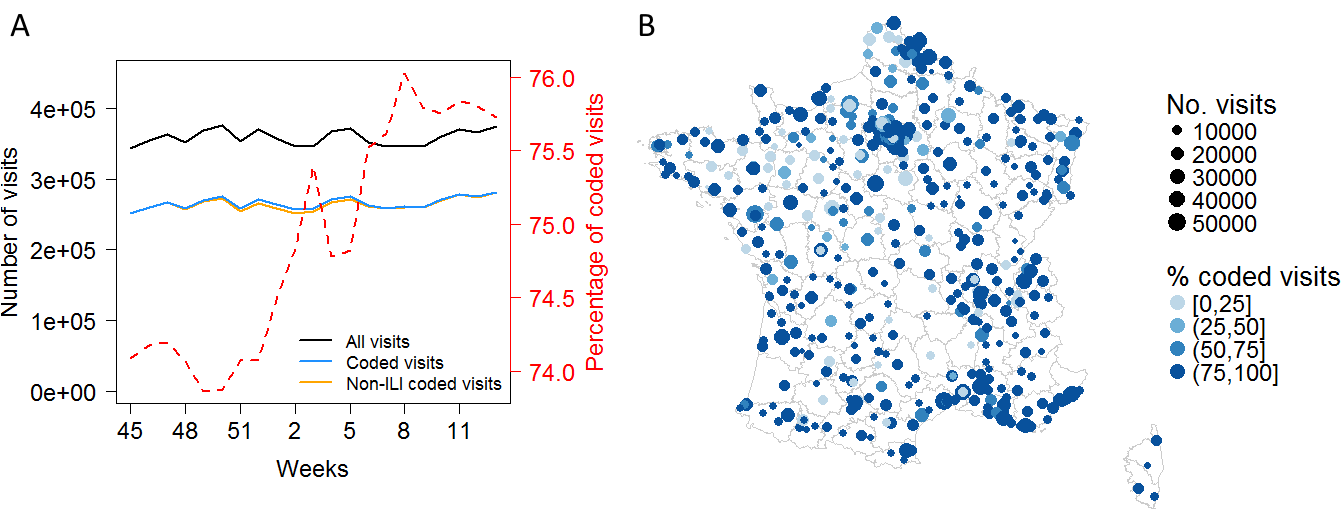


**Figure S1:** Distribution of the percentage of coded visits (A) by week and (B) by emergency departments, over the study period (November 7, 2016 to April 2, 2017) in metropolitan France. The percentage of coding increased over time, from 74 % at the beginning of the 2016-2017 flu season to 76 % at the end. It also varied between ED: the median percentage of coding was 88 %, with an inter-quartile range between 53.5-97.5 %.

# 3. Methods for the Bayesian geostatistical model

We used the R-INLA package to implement the Bayesian model-based geostatistics (MBG) method (www.r-inla.org). Model theory and implementation in R have been thoroughly described.^1–4^ Here, we provide a summary of key features and the R code for the model.

## 3.1. Model theory

In our modelling framework, the spatial process is a Gaussian random field (GRF), characterized by a spatial covariance matrix. One disadvantage of the modeling approach involving the spatial covariance function is the computational cost required for algebra operations with dense covariance matrices.

A computationally effective alternative consists in representing the continuous spatial process (the GRF) using a discretely indexed spatial random process (a Gaussian Markov random field (GMRF)). Indeed, GMRFs are characterized by sparse precision matrices, which make numerical methods involving GMRFs computationally efficient.

This approach has been proposed by Lindgren et al.^5^ and is based on the SPDE (stochastic partial differential equation), whose exact and stationary solution is a GRF with Matérn covariance function. Lindgren et al. showed that an approximated solution to the SPDE can be obtained using a finite element method, where the infinite dimensional GRF is replaced with a finite basis function representation:

$$U\left( s \right)= \sum_{k=1}^{m} \varphi_{k}(s)\tilde{U}_{k}$$

The $\tilde{U}_{k}$’s are zero mean Gaussian distributed weights and the $\varphi_{k}$’s are piece-wise linear basis functions defined on a triangulation of the domain, i.e. a subdivision into non-intersecting triangles with *m* nodes. The figure S2 shows the triangulation of France that was used for this analysis.

In R, the model is fitted using the inla() function of the R-INLA package. The results include summaries and marginal posterior densities of each parameter of the model: the regression parameters, each element of the latent field and all the hyperparameters.

When the objective is to make predictions on a fine grid to get high-resolution maps, the first approach is to make predictions jointly with the estimation process. However, this can be a computationally expensive task when the number of pixels in the grid is large. When the posterior marginal distributions are not necessary, a less computationally heavy solution consists in projecting the latent field – estimated at the mesh vertices – onto the grid locations, after the estimation process. Since only the posterior mean and standard deviation were necessary in our study, we chose the latter option.

Every week was fitted independently. It is also theoretically possible to fit a spatio-temporal model by adding a temporal component, to take into account the temporal correlation between observations. This could help to reduce uncertainty, especially for small EDs. However, this would probably increase computation times considerably.


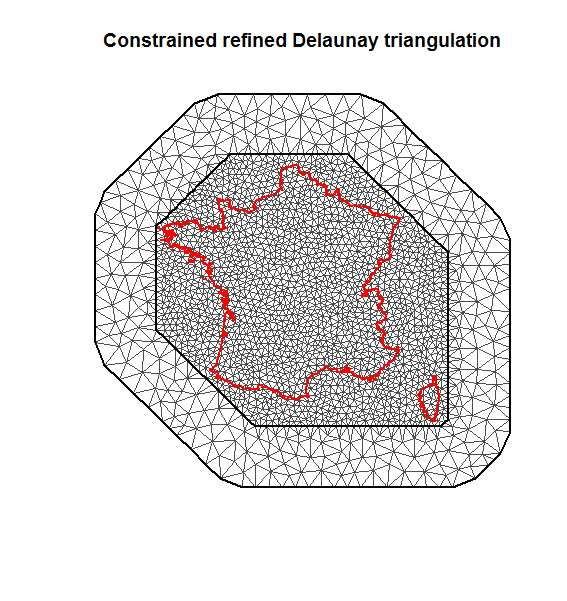


**Figure S2:** Triangulation of metropolitan France used to build the spatial model (about 2300 vertices). For the inner domain, we used a maximum edge of 20 km, which provided a good trade-off between the accuracy of the GMRF representation and computational costs.^1^

## 3.2. Priors

One crucial step in Bayesian modelling that can deter non-specialists to implement these models is the choice of prior distributions. Choosing the prior distributions was proven to be particularly important for Matérn GF, where identifiability issues can arise, but remains a challenge.^6,7^ The Penalised Complexity (PC) prior framework recently developed by Simpson et al.^8^ and Fuglstad et al. for the Matérn GF^6^ now provides a practical approach to build priors for hierarchical additive models, that we chose to follow in this study. Their approach allows the user to control the priors of the parameters by supplying knowledge of the scale of the problem, and make the priors easier to interpret, with a clear meaning.

Fuglstad et al. constructed a weakly informative joint prior for the range and the marginal variance of Matérn GF, using the PC prior framework. The general idea behind PC priors is that simpler models are preferred, i.e. shrinking stationary GRFs towards infinite range and zero variance. Hyperparameters of PC priors are set by selecting the lower tail probability that the range will be below a certain value, and by selecting the upper tail probability that the marginal standard deviation will exceed a certain value. For the present analysis, we chose to set the hyperparameters through $P\left( r<20 \right)=0.01$and$P\left( \sigma>10 \right)=0.01$. We thus considered unlikely a range below 20 km and a standard deviation greater than 10. We also used a PC prior for the precision parameter of the random error, with hyperparameter set to satisfy$P\left( \sigma>1 \right)=0.01$, considering a standard deviation greater than 1 unlikely for the noise.

## 3.3. R code

We present below the R code for the model specification using the R-INLA package.

################################################################################

# store the coordinates

coords <- as.matrix(my.data[,c("X","Y")])/1000

# create the mesh

mesh<-inla.mesh.2d(loc=coords,max.edge=c(20,100),cutoff=20, offset = c(50, 250))

#plot(mesh)

# create the SPDE object (with PC-priors)

spde <- inla.spde2.pcmatern(mesh=mesh, alpha=2, prior.range=c(20, 0.01),

prior.sigma=c(10, 0.01), constr=T)

# create the projector matrix (to project the random field modeled at the mesh nodes)

A.est <- inla.spde.make.A(mesh=mesh, loc=coords)

# create list of indexes

s.index <- inla.spde.make.index(name="spatial.field", n.spde=spde$n.spde)

my.data$loc.index<-1:nrow(my.data)

# stack all together for the estimation process

stack.est <- inla.stack(data=list(y=my.data$cases,Ntrials=my.data$total),

A=list(A.est,1),

effects=list(c(s.index, list(intercept=1)),

list(loc.index=my.data$loc.index)),

tag="est")

# create a projector matrix for the prediction step and stack all pieces together

A.pred <- inla.spde.make.A(mesh=mesh)

mesh.index <- nrow(my.data)+(1:mesh$n)

stack.pred.response <- inla.stack(data=list(y=NA),

A=list(A.pred,1),

effects=list(c(s.index, list(intercept=1)),

list(loc.index=mesh.index)),

tag="pred.response")

# join the two linear predictors

join.stack <- inla.stack(stack.est, stack.pred.response)

# extract the indexes

index.est <- inla.stack.index(join.stack,tag="est")$data

index.pred.response <- inla.stack.index(join.stack,tag="pred.response")$data

# define the formula including all the effects

formula <- y ~ -1 + intercept + f(spatial.field, model=spde) +

f(loc.index,model="iid",hyper=list(prec=list(prior="pc.prec",param=c(1,0.01))))

data.stack<-inla.stack.data(join.stack)

# call the inla function with Binomial likelihood

output<- inla(formula,

data=data.stack,

family="binomial",

Ntrials=data.stack$Ntrials,

control.predictor=list(A=inla.stack.A(join.stack),

link=1, compute=TRUE))

# extract estimates for model parameters

output$summary.fixed # intercept

output$summary.hyperpar # hyperparameters

# extract fitted values at the data locations

res.est<-output$summary.fitted.values[index.est,c("mean","sd")]

# create a regular grid of points, where predictions will be computed

grid.res<-2

grid.x <-round((extent(france)[2]-extent(france)[1])/1000/grid.res)

grid.y <-round((extent(france)[4]-extent(france)[3])/1000/grid.res)

pred.grid <- expand.grid(x = seq(extent(france)[1]/1000, extent(france)[2]/1000, length.out = grid.x), y = seq(extent(france)[3]/1000, extent(france)[4]/1000, length.out = grid.y))

# extract the posterior mean and standard deviation of the response variable at the mesh vertices

res.mesh<-output$summary.fitted.values[index.pred.response,c("mean","sd")]

# create a linkage between the mesh and the grid

proj.grid <- inla.mesh.projector(mesh, xlim=range(pred.grid[,1]),

ylim=range(pred.grid[,2]), dims=c(grid.x,grid.y))

# project the response variable from the mesh to the grid

post.mean.pred.grid <- inla.mesh.project(proj.grid, res.mesh$mean)

post.sd.pred.grid <- inla.mesh.project(proj.grid, res.mesh$sd)

################################################################################

# 4. Comparison to Kriging

In geostatistics, the most widely used method, known as Kriging, allows to carry out spatial interpolation or smoothing of observed values, by constructing a linear predictor for unobserved values of a continuous spatial process and estimating the covariance structure of the data with a tool known as the variogram.^9,10^ The empirical variogram represents the averaged semivariance as a function of distance between pairs of points. Typically, when there is spatial autocorrelation in the data, the semivariance (measuring dissimilarity between observations) increases with the distance, until some distance beyond which observations are no longer spatially correlated. A model (spherical, exponential…) is fitted to the points forming the variogram and can then be used to predict values at unsampled locations and create maps of the prediction surface.

As argued by Diggle et al,^11,12^ traditional Kriging is implicitly assuming a linear Gaussian model. However, observations not in agreement with the chosen model can affect badly the variogram estimate. In our case, the observations display asymmetric, skewed distribution (the most common departure from normality in spatial epidemiology) and substantial noise due to unstable rates computed in small populations. When plotting the empirical variogram for the proportion of influenza-coded cases and fitting a Matérn model (covariance structure similar to our MBG model), we could thus (wrongly) conclude that there is no spatial dependence in our data (Figure S3).


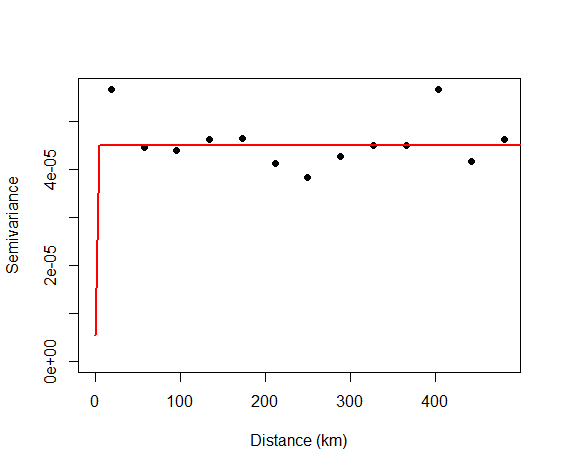


**Figure S3:** Empirical variogram (black points) and best fit using a Matérn model (red line) for the influenza proportion in week 49, 2016.

In these situations where the stochastic variation in the data is known to be non-Gaussian (which is often the case in spatial epidemiology), trans-Gaussian Kriging can be used. This method consists of applying Kriging after a marginal non-linear transformation of the data, such as the log transform for count data.^9^ We log-transformed the data and fitted a Matérn model to the variogram, using the gstat package in R. Figure S4 shows the variogram fit for the three weeks detailed in the main article and the resulting prediction surface maps.


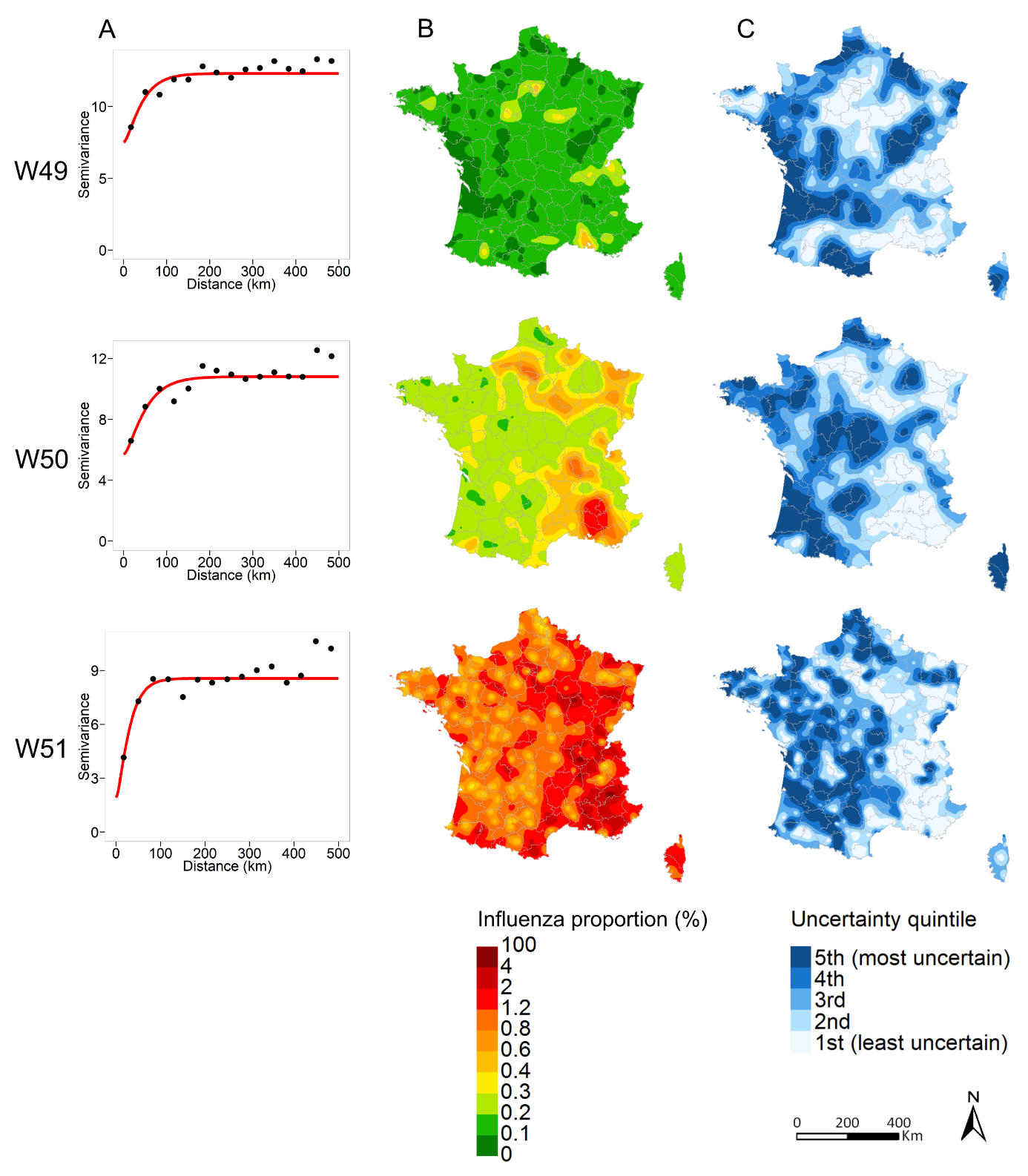


**Figure S4:** Kriging results for weeks 49, 50 and 51, 2016. (A) Empirical variogram of the log-transformed proportion of influenza-coded cases (black points) and best fit using a Matérn model (red line). (B) Predicted influenza proportion on the 2x2km grid. (C) Relative uncertainty associated with the predicted influenza proportion, quantified using the coefficient of variation, and ordered into quintiles such that areas in quintile one have the smallest uncertainty and quintile five the largest. Greys borders delimit administrative districts (N=96).

At first glance, the Kriging maps look very different from the MBG maps. To properly compare the Kriging results to the MBG results, we computed the same statistics used for the MBG model assessment. First, to assess the accuracy of the spatial predictions, we compared the observed influenza proportion at the district level (N=96) to the grid predictions averaged by district, using Pearson’s correlation coefficient, the mean error and the mean absolute error. Then, we compared the predictions made when the semivariogram was fitted to the full dataset, to the predictions made when each observation was, in turn, removed from the data (leave-one-out predictions). Table S1 shows the different statistics used for model assessment and Figure S5 shows the two corresponding scatterplots for the Kriging method (the scatterplots for the MBG method are given in the main article). Overall, the MBG was performing better than the Kriging method. In particular, the Kriging predictions at the district level underestimated influenza activity (mean error of -0.25% in units of the proportion of influenza-coded cases, 15 times higher than the MBG mean error). And the leave-one out predictions at ED locations showed that the MBG model was better at predicting the smoothed influenza proportion in an unsampled location.

**Table S1:** Statistics used for comparing the performance of Kriging to model-based geostatistics (MBG)

|  | **MBG** | **Kriging** |
| --- | --- | --- |
| **Predictions at the district level** |  |  |
| Pearson’s r | 0.80 | 0.69 |
| Mean error | -0.00016 | -0.00248 |
| Mean absolute error | 0.0032 | 0.0042 |
| **Leave-one-out predictions at ED locations** |  |  |
| Pearson’s r | 0.98 | 0.84 |
| Mean error | 0.0007 | -0.0003 |
| Mean absolute error | 0.0012 | 0.0018 |

**
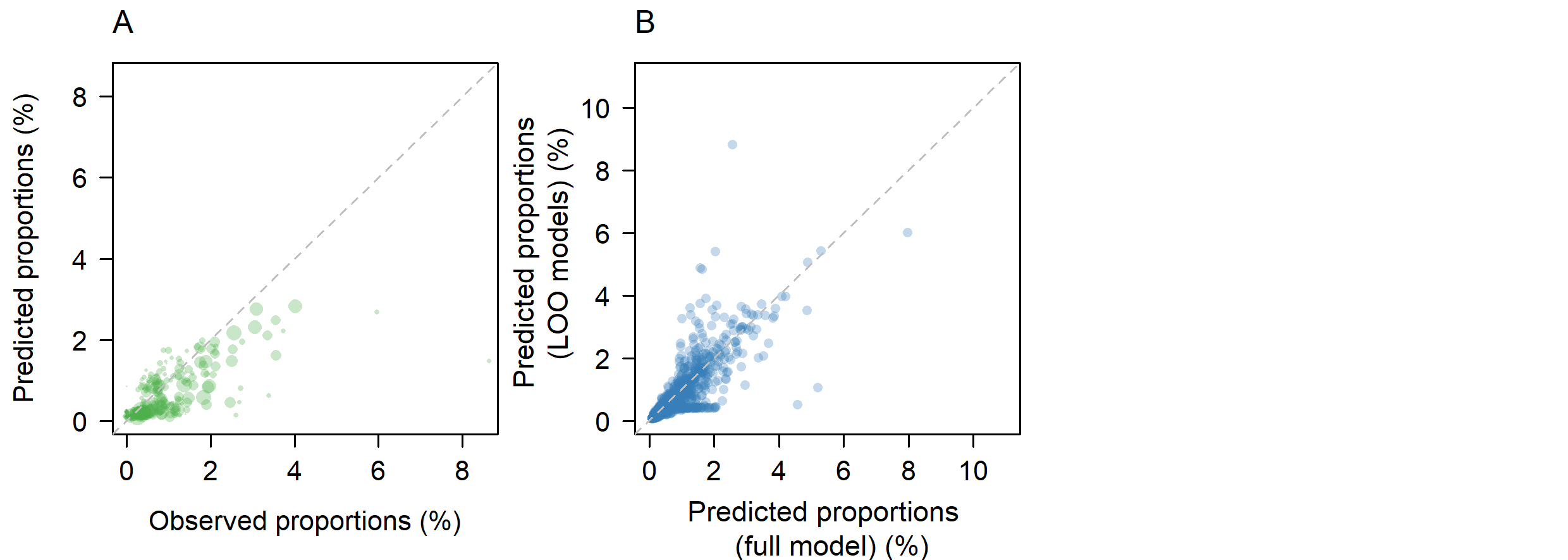
**

**Figure S5:** Kriging method assessment for weeks 49 to 51, 2016. (A) Scatterplot of predicted and observed proportions of influenza-coded cases, averaged at the district level. (B) Scatterplot of predictions using the full dataset and the leave-one-out predictions at the ED locations. Point size is weighted by the number of all coded visits. The dashed line is the bisector.

Finally, we compared the prediction uncertainty estimates between the two methods. We showed that the coefficients of variation in the Kriging method were systematically lower than in the MBG method (Figure S6), suggesting that Kriging may under-estimate the uncertainty in the predictions. This was an expected result since Kriging does not account fully for inherent uncertainties, such as those arising from unstable rates in small populations and those associated with estimating the variogram parameters.^13,14^


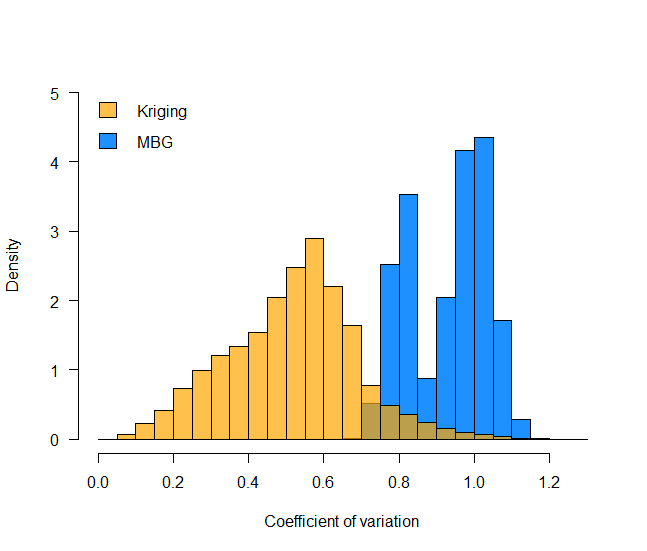


**Figure S6:** Distribution of the coefficient of variation (relative uncertainty) for the grid predictions from Kriging and MBG methods for weeks 49 to 51, 2016.

To overcome these issues associated with standard Kriging, we chose to use model-based geostatistics, a different (though related) statistical framework. Other alternatives have been proposed, such as using Poisson Kriging^13^ or assessing variogram uncertainty in a Bayesian framework.^15^

# 5. Weekly maps for the 2016-2017 season

This section presents the maps resulting from our geostatistical model during the whole study period, from week 45, 2016 (Nov 7 – Nov 13) to week 13, 2017 (March 27 – April 2). Each line represents one week. Left panel: Observed proportion of influenza-coded cases at each ED locations. Central panel: Posterior mean of predicted proportion on the 2x2km grid. Right panel: Relative uncertainty associated with the predicted proportion, quantified using the coefficient of variation, and ordered into quintiles such that areas in quintile one have the smallest uncertainty and quintile five the largest. Greys borders delimit administrative districts (N=96).

**Legend**

**
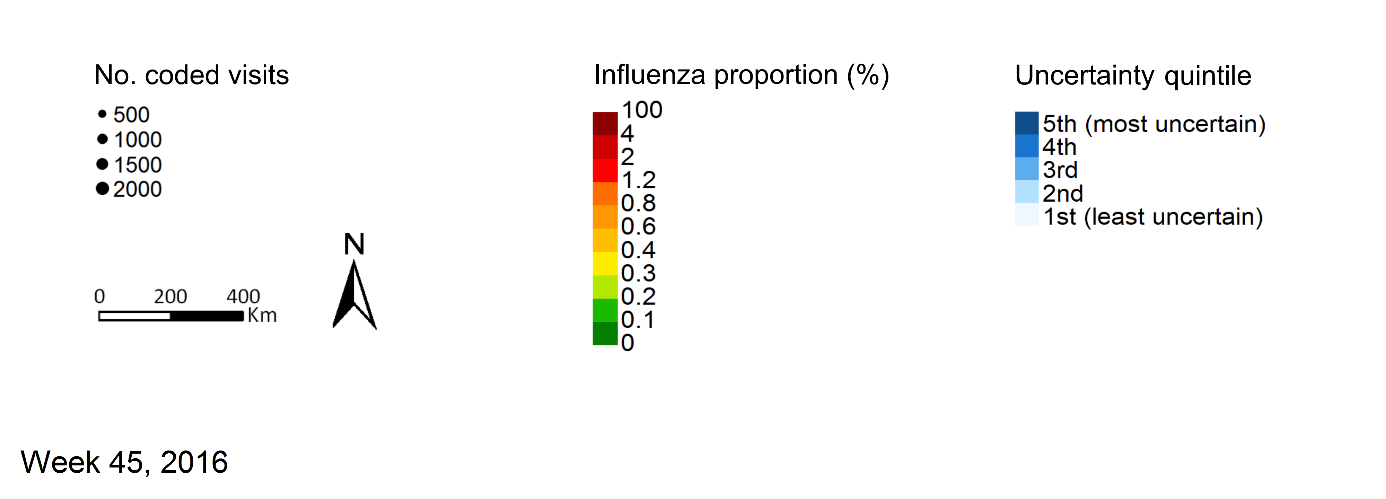
**


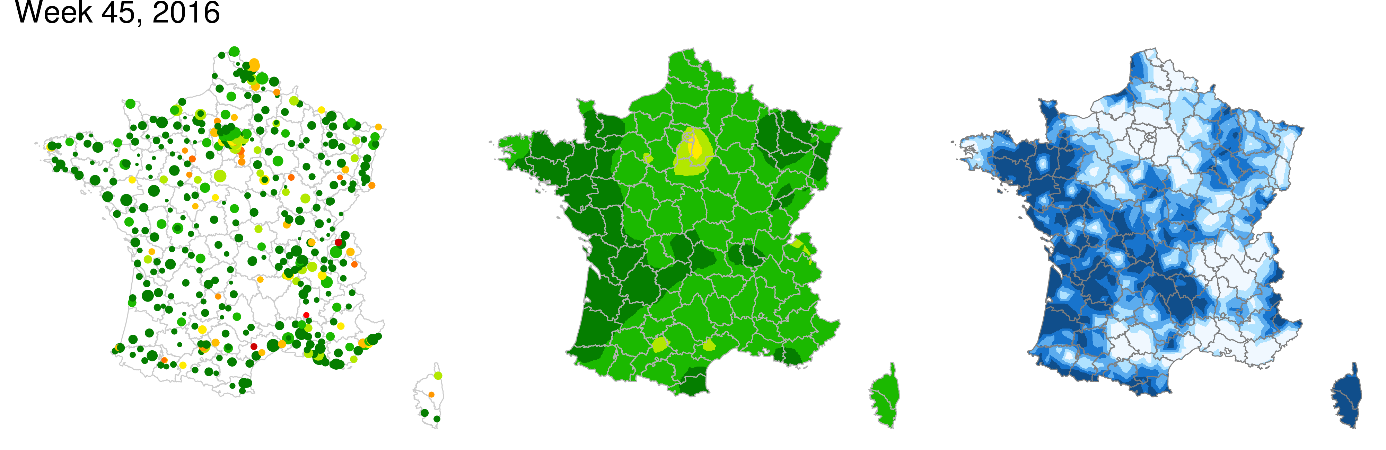

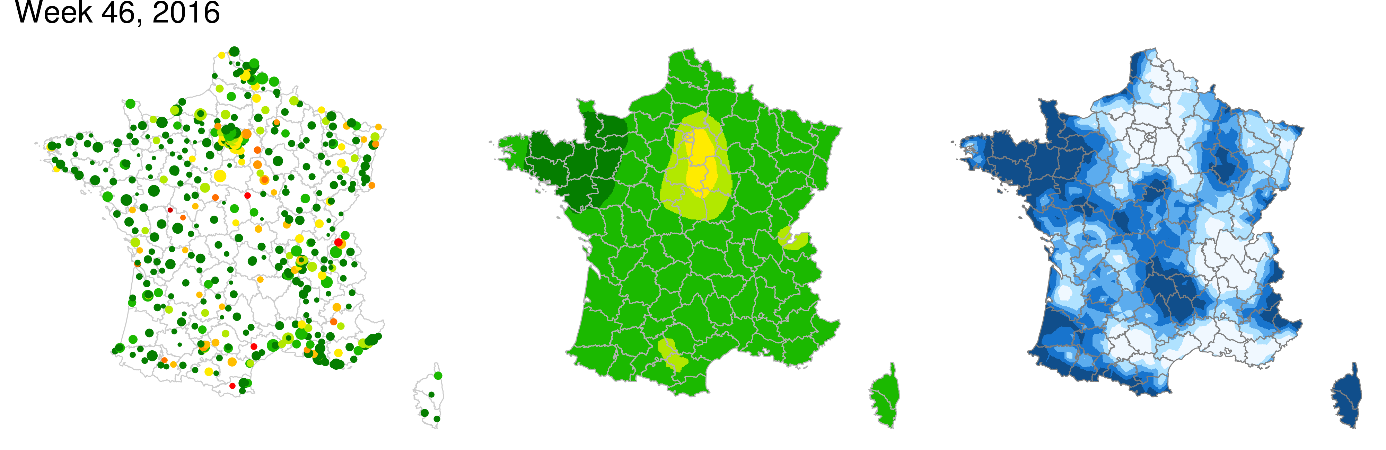

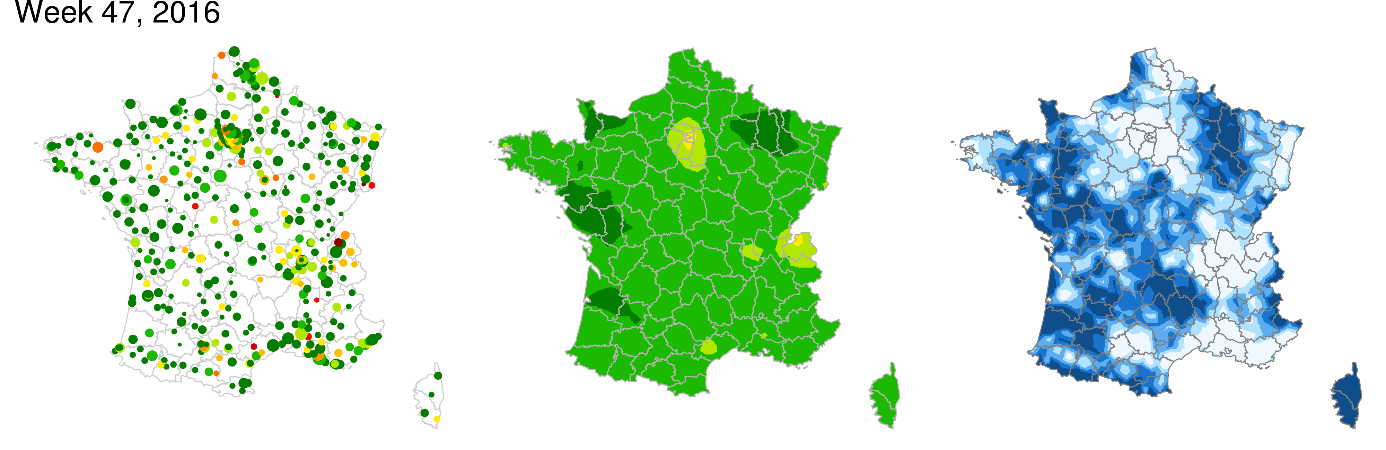

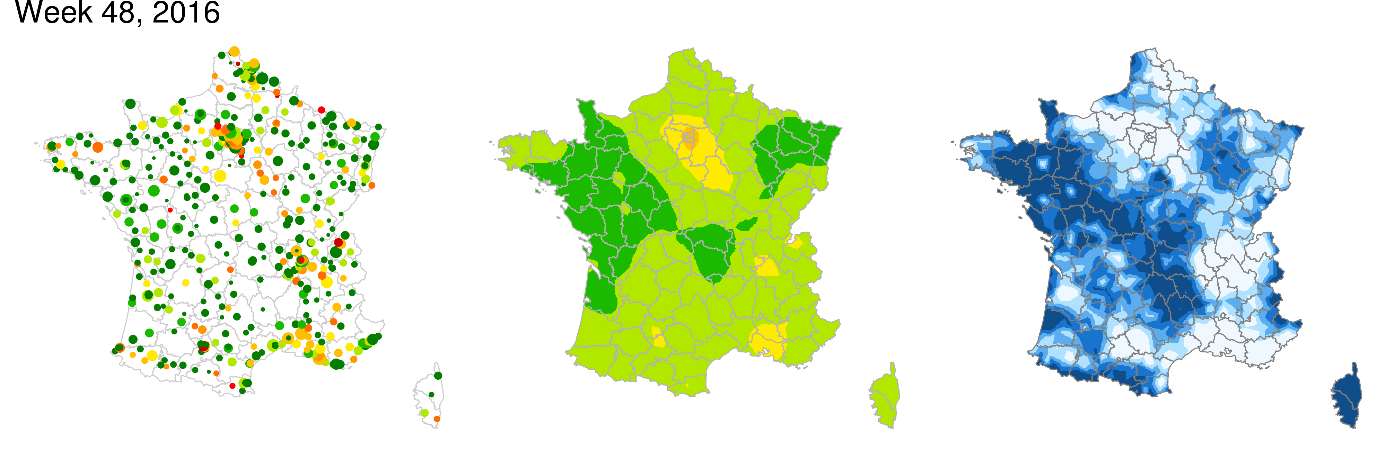

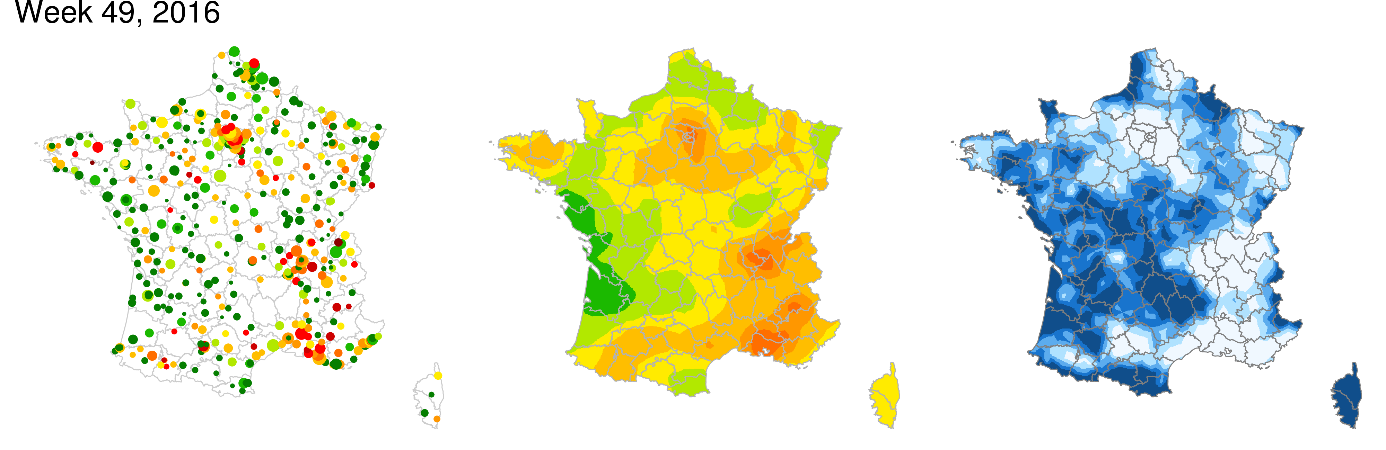

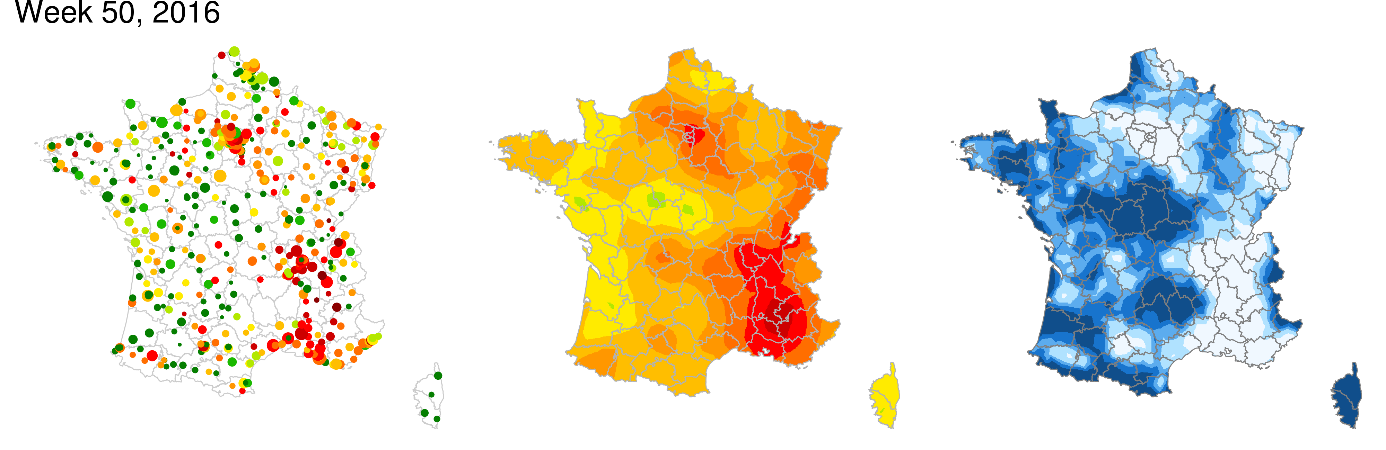

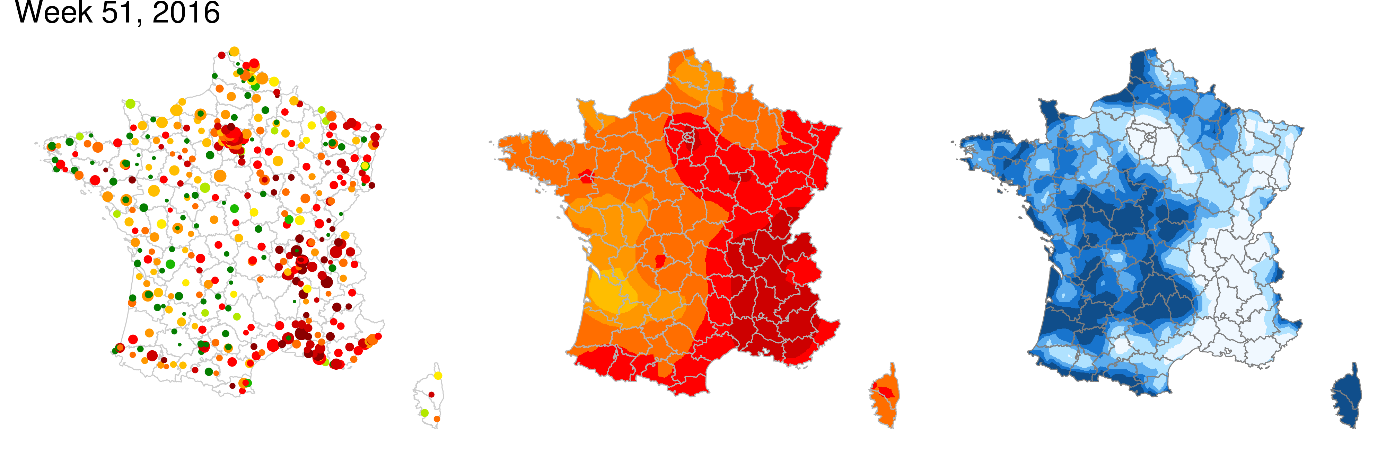

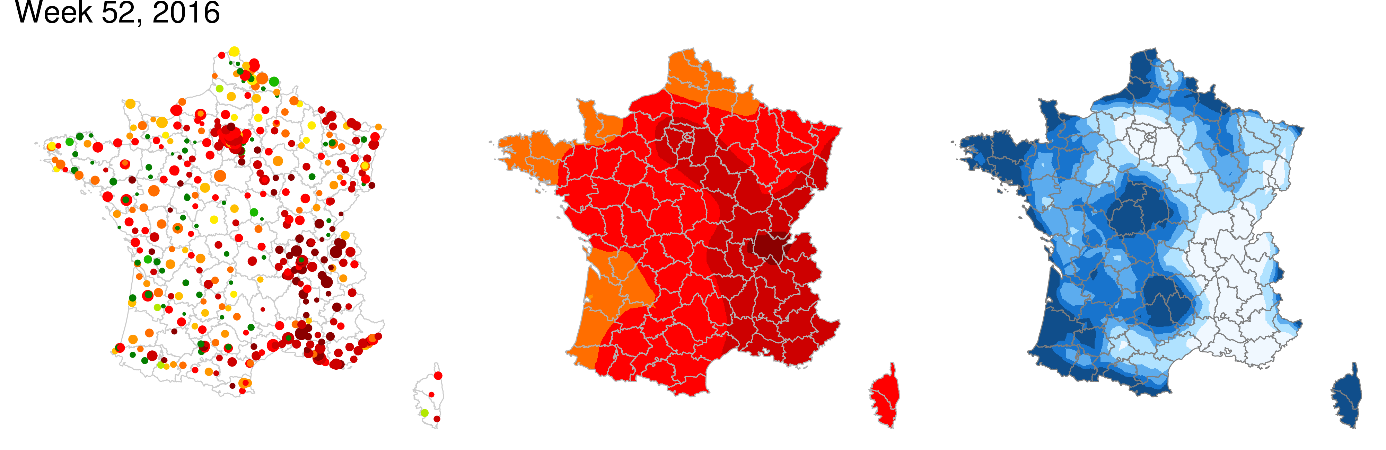

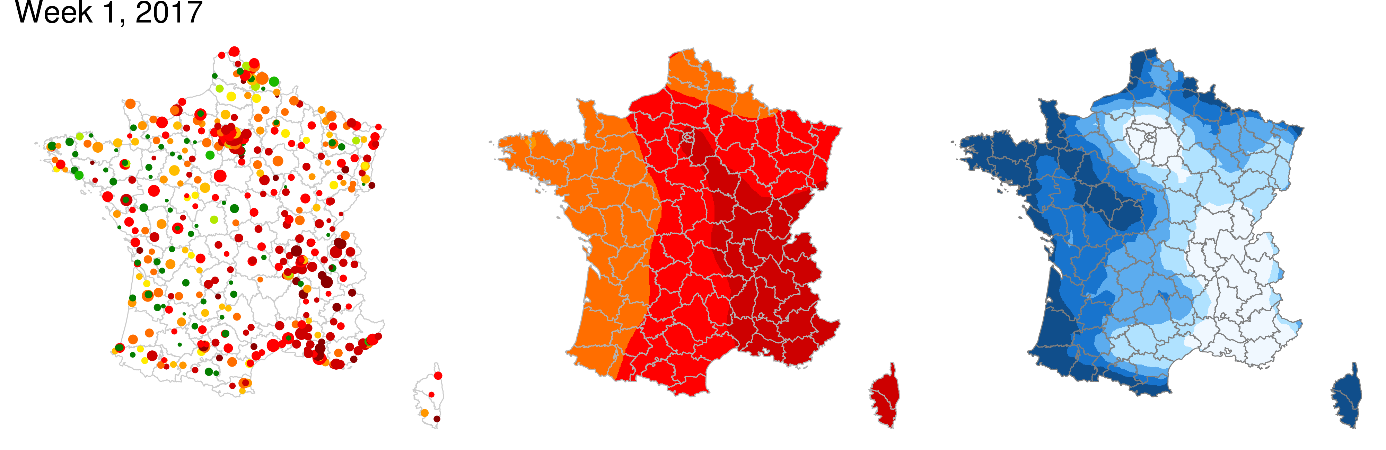

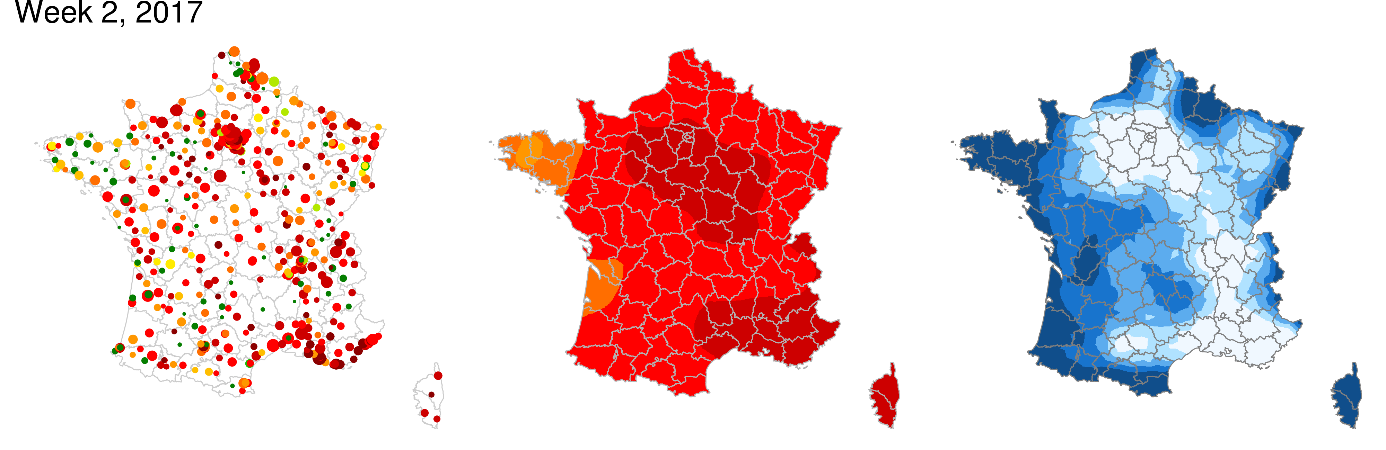

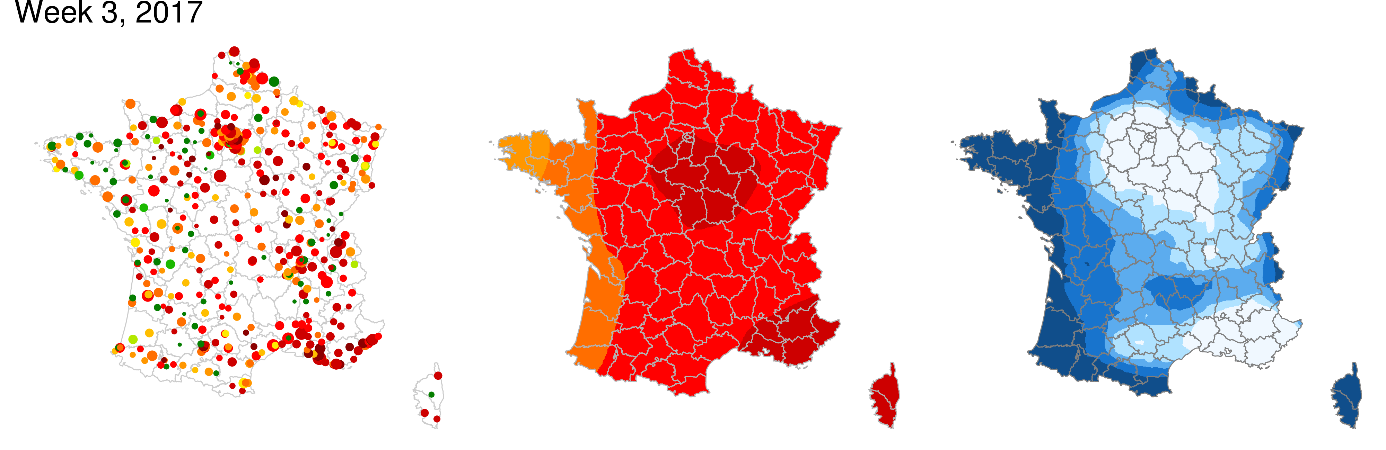

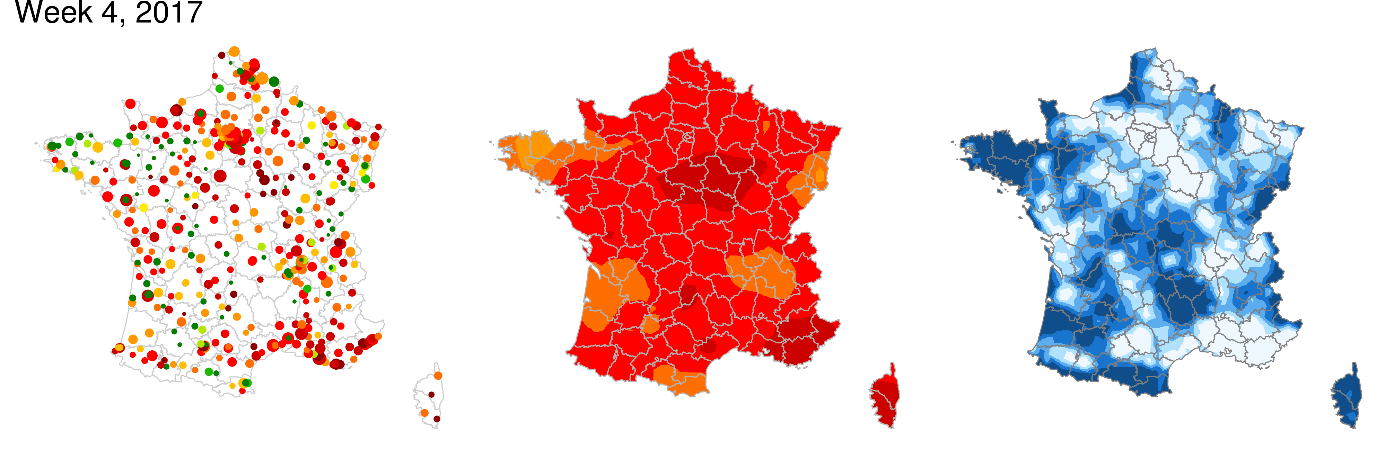

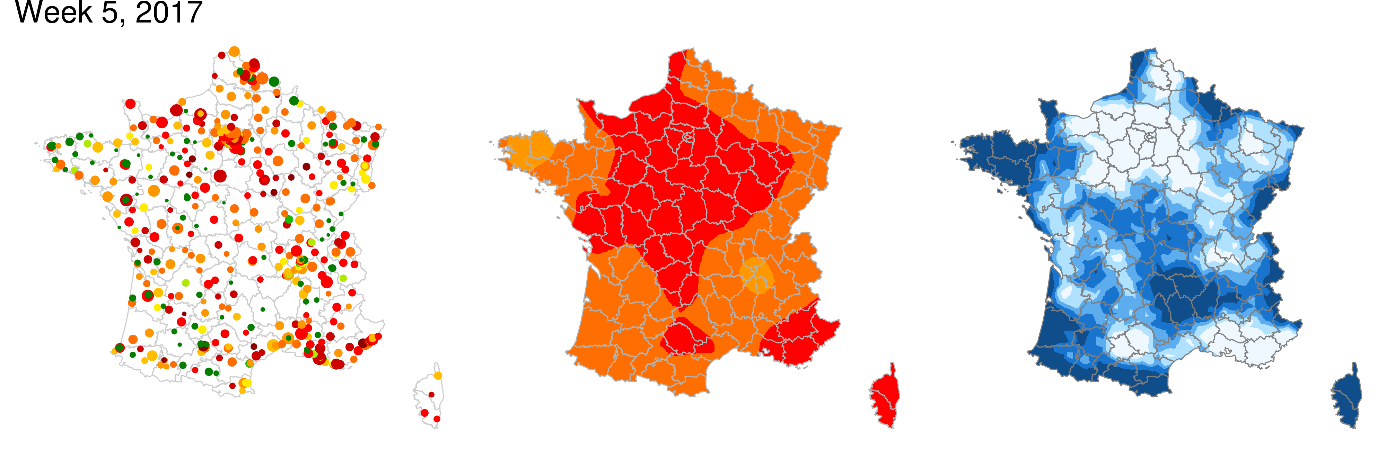

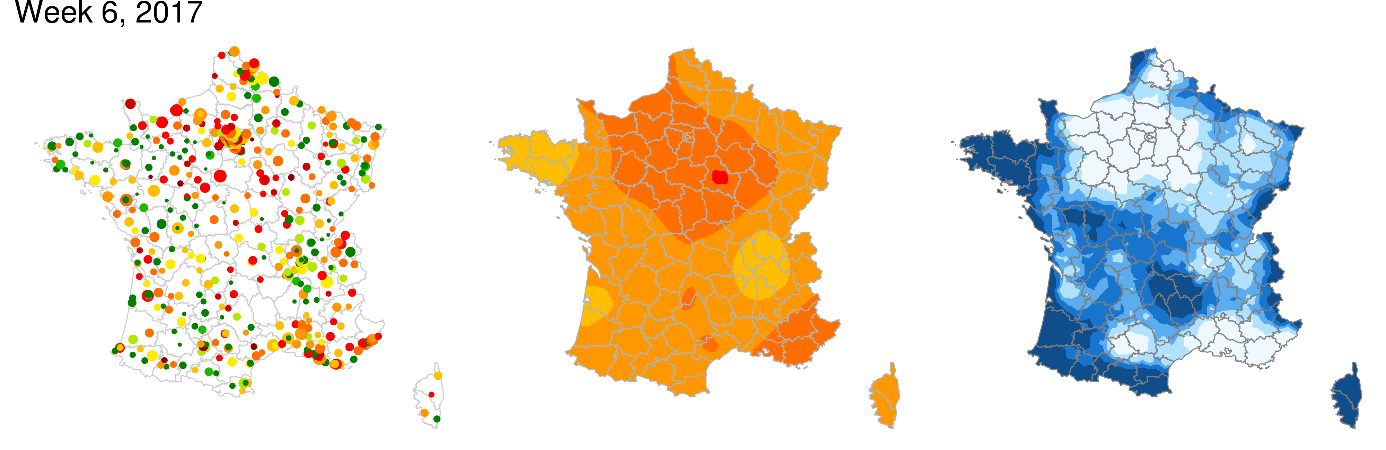

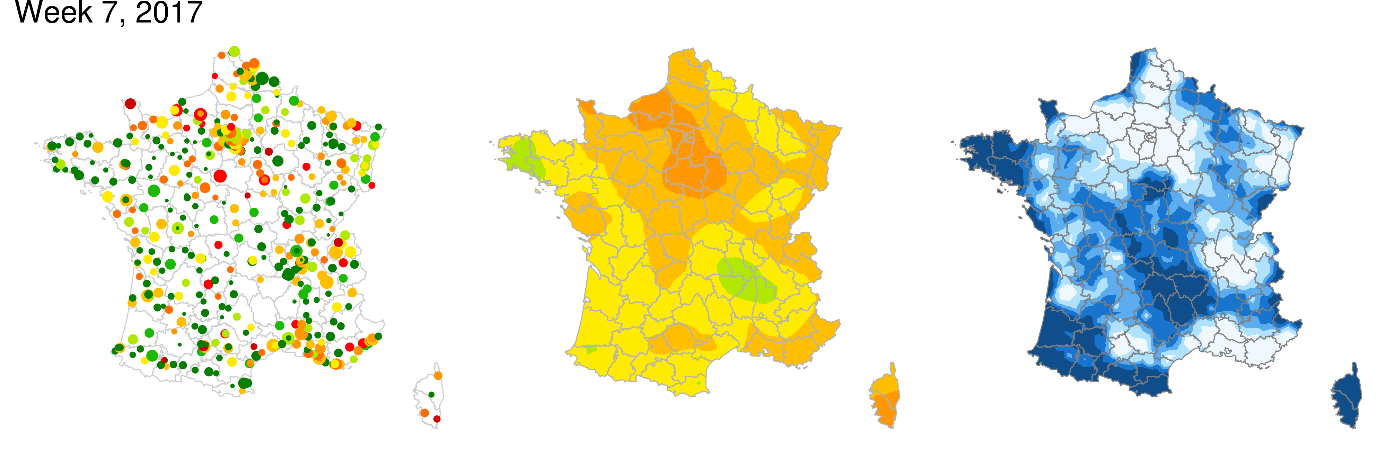

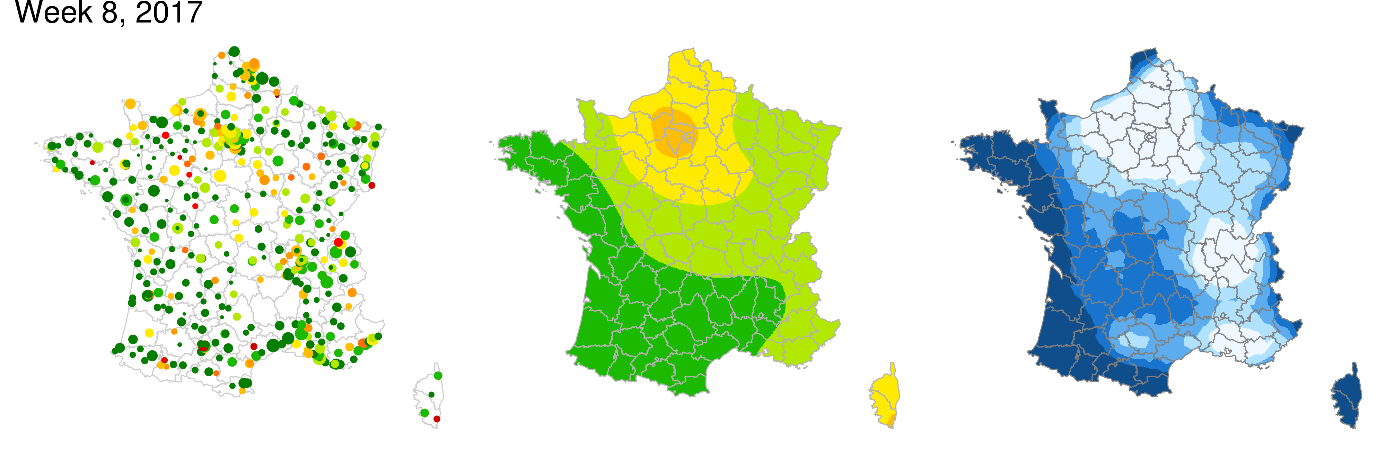

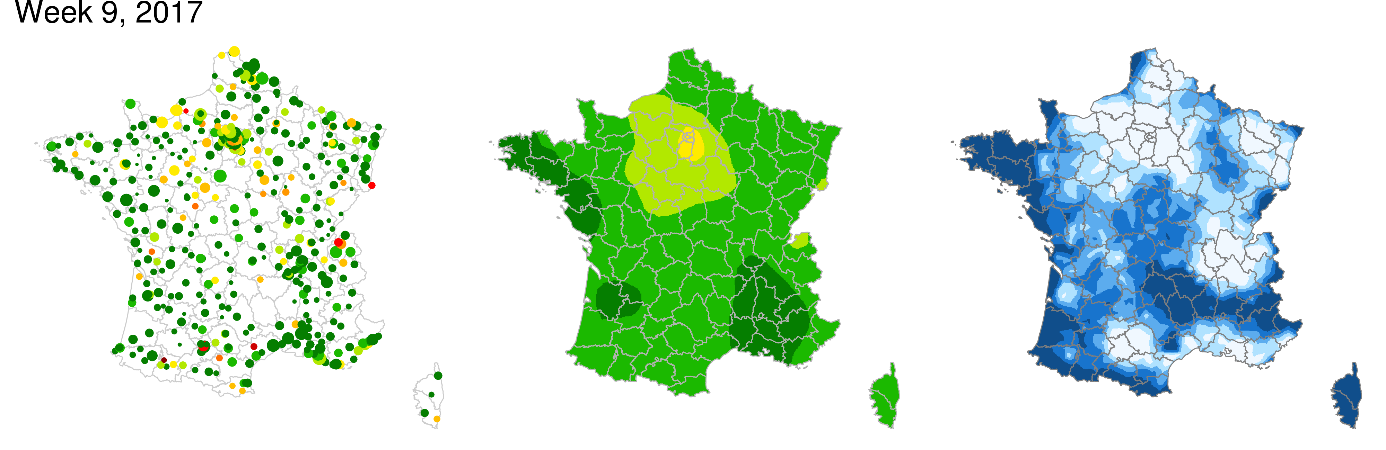

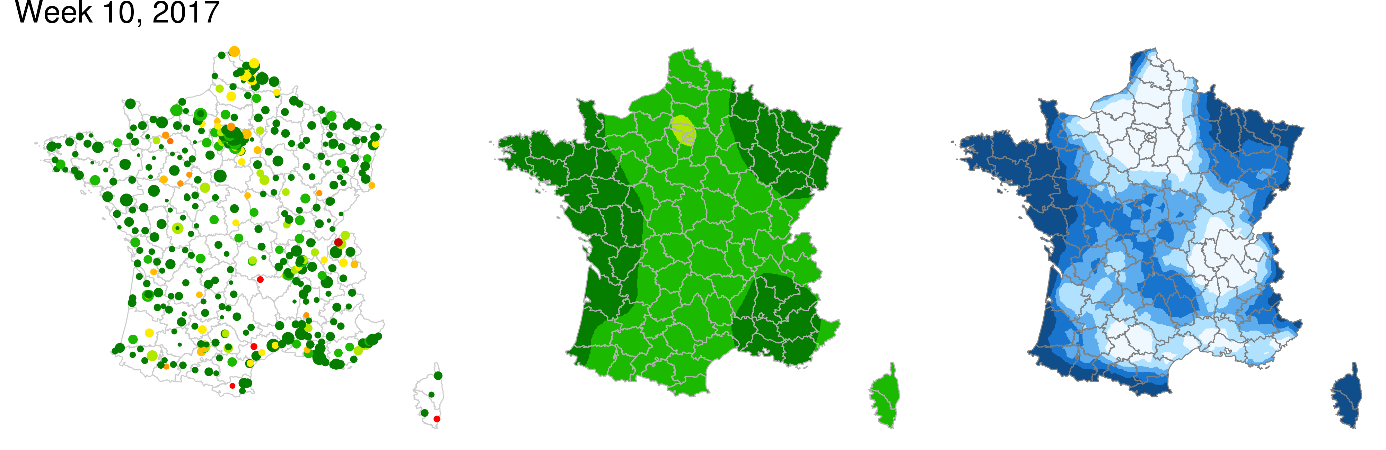

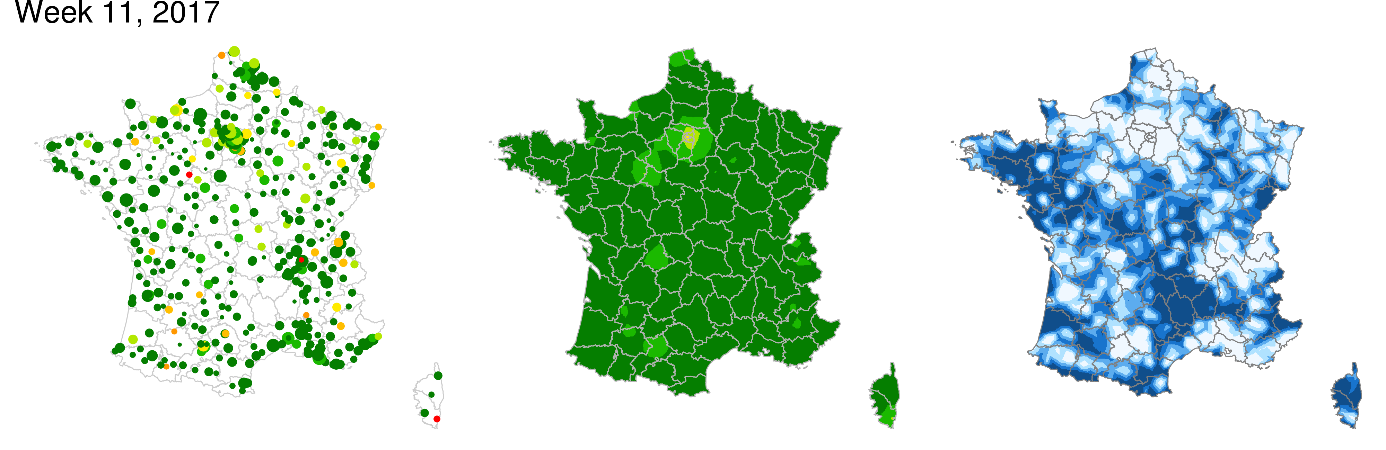

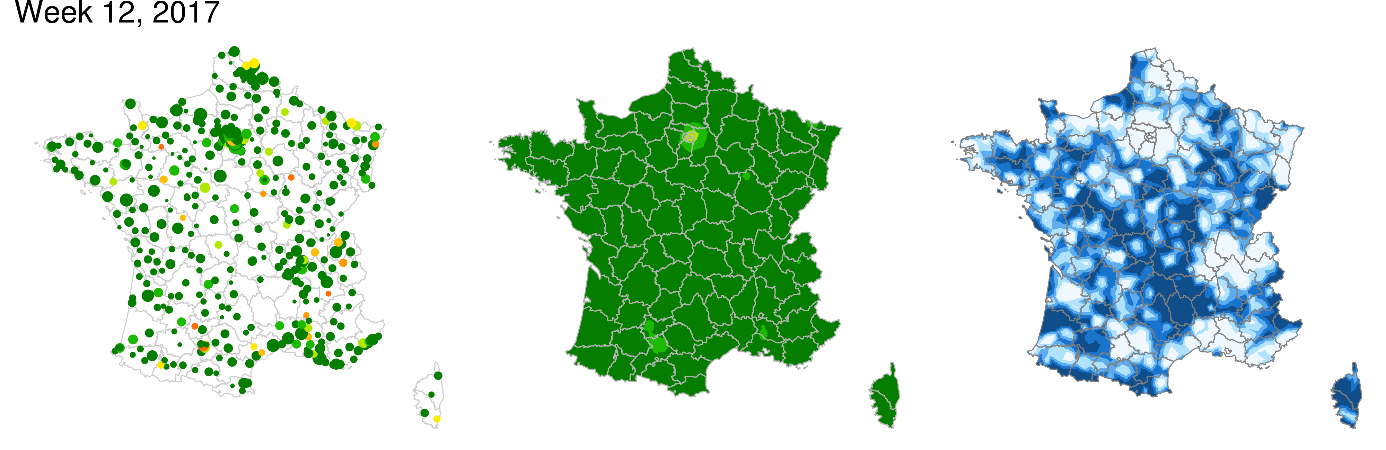

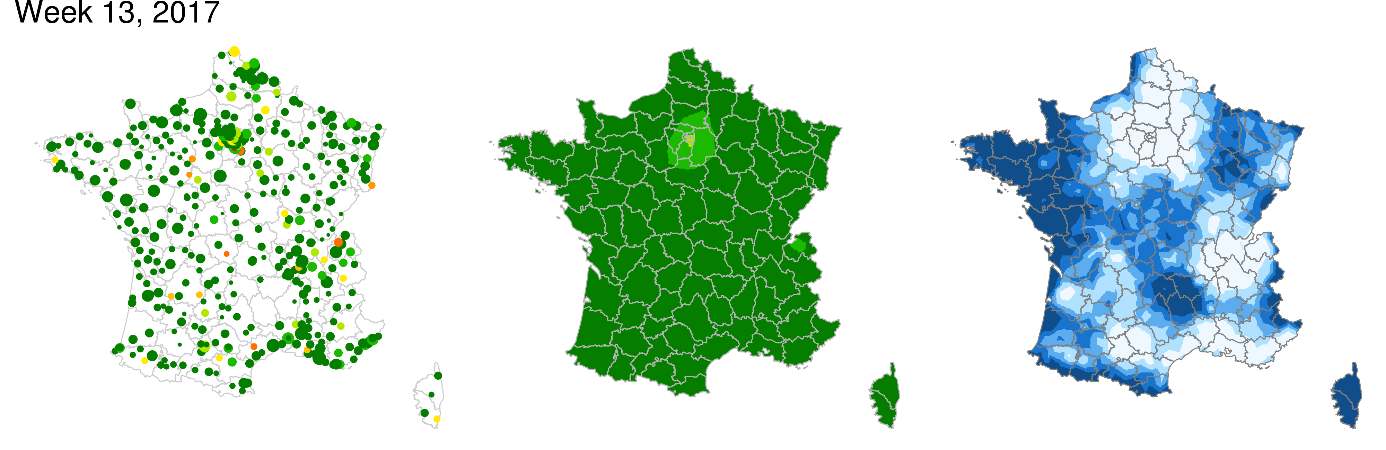


# 6. References

1. Blangiardo M, Cameletti M. Spatial and Spatio-Temporal Bayesian Models with R-INLA. Chichester, West Sussex, UK: John Wiley and Sons, Inc; 2015. 308 p.

2. Krainski ET, Lindgren F, Simpson D, Rue H. The R-INLA tutorial on SPDE models. Available at http://www.math.ntnu.no/inla/r-inla.org/tutorials/spde/html/(cited 20 June 2017)

3. Martins TG, Simpson D, Lindgren F, Rue H. Bayesian computing with INLA: New features. Comput Stat Data Anal 2013;67:68-83.

4. Rue H, Martino S, Chopin N. Approximate Bayesian inference for latent Gaussian models by using integrated nested Laplace approximations. J R Stat Soc Ser B Stat Methodol 2009;71:319-392.

5. Lindgren F, Rue H, Lindström J. An explicit link between Gaussian fields and Gaussian Markov random fields: the stochastic partial differential equation approach: Link between Gaussian Fields and Gaussian Markov Random Fields. J R Stat Soc Ser B Stat Methodol 2011;73:423-498.

6. Fuglstad G-A, Simpson D, Lindgren F, Rue H. Constructing Priors that Penalize the Complexity of Gaussian Random Fields. J Am Stat Assoc 2018.

7. Ingebrigtsen R, Lindgren F, Steinsland I. Spatial models with explanatory variables in the dependence structure. Spat Stat 2014;8:20-38.

8. Simpson D, Rue H, Riebler A, Martins TG, Sørbye SH. Penalising Model Component Complexity: A Principled, Practical Approach to Constructing Priors. Stat Sci 2017;32:1-28.

9. Cressie NAC. Statistics for Spatial Data. New York: John Wiley & Sons, Inc; 2013.

10. Matheron G. Principles of geostatistics. Econ Geol 1963;58:1246-1266.

11. Diggle PJ, Tawn JA, Moyeed RA. Model-based geostatistics. J R Stat Soc Ser C Appl Stat 1998;47:299-350.

12. Diggle PJ, Ribeiro PJ. Bayesian Inference in Gaussian Model-based Geostatistics. Geogr Environ Model 2002;6:129-146.

13. Ali M, Goovaerts P, Nazia N, Haq MZ, Yunus M, Emch M. Application of Poisson kriging to the mapping of cholera and dysentery incidence in an endemic area of Bangladesh. Int J Health Geogr 2006;5:45.

14. Pullan RL, Sturrock HJW, Soares Magalhães RJ, Clements ACA, Brooker SJ. Spatial parasite ecology and epidemiology: a review of methods and applications. Parasitology 2012;139:1870-1887.

15. Marchant BP, Lark RM. The Matérn variogram model: Implications for uncertainty propagation and sampling in geostatistical surveys. Geoderma 2007;140:337-345.
